# Optogenetic modulation of peripheral nociceptive neurons with biocompatible optoelectronic implants

**DOI:** 10.1002/btm2.70034

**Published:** 2025-06-26

**Authors:** Paul Chu Sin Chung, Valentina Paggi, Marie Pertin, Guylène Kirschmann, Elena A. Konnova, Frédéric Michoud, Ivan Furfaro, Bernard L. Schneider, Stéphanie P. Lacour, Isabelle Decosterd

**Affiliations:** ^1^ Pain Center, Department of Anesthesiology Lausanne University Hospital (CHUV) Lausanne Switzerland; ^2^ Laboratory for Soft Bioelectronic Interfaces Neuro‐X Institute, Ecole Polytechnique Fédérale de Lausanne (EPFL) Switzerland; ^3^ Ecole Polytechnique Fédérale de Lausanne (EPFL) Brain Mind Institute Switzerland; ^4^ Bertarelli Foundation Gene Therapy Platform Ecole Polytechnique Fédérale de Lausanne (EPFL) Switzerland; ^5^ Department of Fundamental Neurosciences, Faculty of Biology and Medicine University of Lausanne Lausanne Switzerland

**Keywords:** ArchT, dorsal root ganglia, nociception, optoelectronic implant, optogenetic, pain

## Abstract

Hyperexcitability of peripheral sensory neurons plays a critical role in the development and maintenance of chronic pain. Pharmacological analgesics used in clinics reduce neuronal activity. They often come with non‐negligible side effects. Optogenetic approaches can modulate neuronal activity and are attracting growing interest for therapeutic uses, but the delivery of light in different parts of the body requires the development of specific optoelectronic interfaces. We designed and produced a microfabricated optoelectronic implant to deliver yellow light (559 nm) onto the sciatic nerve. We have surgically implanted the device in transgenic mice expressing the yellow light‐sensitive inhibitory archaerhodopsin (ArchT) in nociceptive neurons. Yellow light induced a significant reduction in the responses of the nociceptive neurons and curbed the behavioral responses to noxious mechanical and thermal stimuli. Remarkably, the yellow light‐related inhibition did not alter the behavioral responses evoked by innocuous mechanical stimulation or by intense inflammation. The optoelectronic implants showed reliable and reproducible opto‐electrical performance. For stimulation parameters used in vivo (3.3 V, 60–80 mW/mm^2^, 20 s train pulses, 1 Hz, 80% duty‐cycle, and an inter‐train interval of 1 s), limited temperature increase was measured in an environment mimicking neural tissue surrounded by muscle and fat. Similarly, the basal sensitivity of the implanted mice remains comparable to non‐implanted mice, suggesting a safe integration of the soft electronic device. Our study confirmed that optoelectronic implants tailored to the sciatic nerve can provide specific light spectra and intensities at adequate levels for the optogenetic actuator to trigger significant electrophysiological and behavioral responses in pain perception.

**Translational Impact Statement**

Our study demonstrates a novel optoelectronic implant designed to modulate pain by inhibiting nociceptive neurons in the sciatic nerve using light. This soft, biocompatible implant, with miniaturized LEDs, offers precise, wireless control of neuronal activity without pharmacological intervention. By reducing sensitivity to painful stimuli while preserving normal sensory function, this innovative approach holds significant translational potential for treating chronic pain conditions. This technology represents a promising step toward the development of long‐term, non‐invasive pain management strategies, with potential applications in human clinical therapies.

## 1 INTRODUCTION

Millions of people worldwide suffer from chronic pain, including 20% of the population in the USA and EU.^1^ Current treatments are challenging and often accompanied by significant side effects, which notably affect patients' daily lives.^2^ In recent decades, a deep and exhaustive understanding of the neurobiological mechanisms involved in pain chronification has emerged.^3^, ^4^ Sensitization processes have been extensively studied and described as long‐term molecular and cellular adaptations occurring within both the peripheral^5^ and central nervous system.^6^, ^7^

Primary sensory neuron hyperexcitability leads to^8^ abnormal peripheral inputs responsible for spontaneous pain and also contributes to the mechanisms involved in central sensitization and the establishment of long‐lasting hypersensitivity.^9^ The complexity and diversity of pathological processes may account for the limited efficacy of treatments targeting specific pathways.

An alternative emerging strategy is to directly modulate the electrical activity of peripheral sensory neurons,^10^ thereby cutting off the peripheral abnormal input.

Optogenetic technology is a non‐pharmacological approach that selectively activates or reduces neuronal activity through light. It is now used as a tool to address scientific questions in pain research and various other neuroscience fields.^11^, ^12^ The inhibitory opsin archaerhodopsin Arch is a proton pump sensitive to yellow light (575 ± 25 nm).^13^, ^14^ Transdermal application of yellow light reduces mechanical and thermal pain sensitivity in mice expressing Arch using viral vector‐based gene therapy. Furthermore, it has been shown to reduce neuropathic pain‐related mechanical and thermal sensitivity in mice after nerve injury.^15^, ^16^ Optogenetic approaches allow controlling a selected cell population in a time‐ and space‐controlled manner. For example, selective expression of Arch in calcitonin gene‐related peptide (CGRP*α*) sensory neurons, combined with transdermal yellow light stimulation, could reverse the mechanical and thermal neuropathic pain‐related hypersensitivity induced by nerve injury.^17^ Similarly, Arch‐induced inhibition of Na_v_1.8^+^ sensory neurons has been shown to reduce hypersensitivity after nerve injury or in inflammatory conditions,^18^ as well as in the lachrymal gland excision LGE –experimental pain model.^19^ These encouraging findings demonstrate the potential of optogenetic approaches for therapeutic applications.

To deliver light on peripheral nerves, miniaturized interfaces made of compliant and biocompatible materials were elaborated in recent years.^20^, ^21^, ^22^ This development has proven efficient in activating the excitatory opsin channelrhodopsin (ChR) and promoting nocifensive behaviors without causing notable inflammation and injuries.^23^, ^24^, ^25^, ^26^ It bypasses the challenging constraints of optic fiber implantation in the nervous system, alleviates physical restrictions for the animals, and provides long‐term stability of the light source. Similar progress was made in the design of refined devices which allow modulating the function of specific organs, such as the stomach^27^ or the bladder.^28^, ^29^ Interestingly, optoelectronic implants on the bladder showed that inhibiting the afferent Na_v_1.8^+^ nociceptive neurons of the bladder, using an Arch opsin, could restore a normal bladder function in experimental cyclophosphamide‐induced cystitis.^29^ Significant improvements in the field have been made by the development of refined devices powered through miniaturized antennas^30^, ^31^ or Bluetooth‐controlled headstage units.^32^ These studies also demonstrated that blue light stimulation applied to the sciatic nerve via the optoelectronic implant could elicit nocifensive behavior in TRPV1‐ChR2 transgenic mice compared to their control littermates. Recently, stretchable hydrogel optical fibers, similar to these peripheral miniaturized optoelectronic devices, have been used to selectively inhibit TRPV1‐expressing inhibitory Halorhodopsin channel neurons, thereby reducing the inflammatory pain phenotype.^33^

Here we report on the development, validation, and effect on pain sensitivity of an optoelectronic implant for the sciatic nerve. Analysis of the physical properties of the optoelectronic implant shows that it can trigger powerful and reliable light stimulation at 559 nm (yellow light) without producing a significant temperature increase under in vivo*‐like* experimental conditions. The implant surgery itself has no unforeseen impact on the basal pain sensitivity in mice. We produced and characterized the SNS‐ArchT transgenic mouse line for further in vivo experiments. Electrophysiological patch‐clamp recordings have confirmed the efficiency of ArchT inhibitory opsin in reducing the excitability of primary sensory neurons. Silencing with yellow light of nociceptive neurons reduced the mice's pain sensitivity to mechanical and thermal noxious stimuli, without altering their response to non‐nociceptive stimuli. Our study offers proof of concept for using optogenetic and soft implantable neurotechnology to remotely inhibit nociceptive neurons on the sciatic nerve.

## 2 MATERIALS AND METHODS

The references of material, reagents, and instruments are listed in the supplemental Table 1.

### 2.1 Study approval

All animal experiments adhered to Swiss Federal Animal Protection Law and were approved by the Committee on Animal Experimentation of the Canton de Vaud, Switzerland (34747/VD3068x2d). The study followed US National Institutes of Health guidelines^34^ and the International Association for the Study of Pain (IASP) guidelines.^35^ All animal studies were conducted following the ARRIVE guidelines. Animals were randomly assigned to experimental groups by another laboratory member, and outcome assessments were performed in a blinded manner. The experimental procedures, including housing conditions and surgical interventions, were standardized to ensure reproducibility and minimize bias.

### 2.2 Animals

Animals were group‐housed in standard conditions with access to food, water, and a 12 h/12 h light/dark cycle. Both male and female mice (50 males, 20 females) aged 5–8 weeks were used. Transgenic mice were generated by crossing *LSL‐ArchT‐eGFP* mice (B6.Cg‐Gt(ROSA)26Sortm40.1(CAG‐aop3/EGFP)Hze/J) with *SNS‐Cre* mice (Tg(Scn10a‐cre)1Rkun),^36^ resulting in conditional ArchT‐eGFP expression in sensory Na_v_1.8^+^ DRG neurons.

### 2.3 Biocompatible optoelectronic sciatic implants

*Micro‐LED array fabrication*. The optoelectronic implants were built as previously described^32^ (see simplified microfabrication process in Supplemental Figure S1). Briefly, a 3 μm‐thick layer of polyimide was spin‐coated on a wafer previously coated with a sacrificial Ti/Al layer (20/100 nm) and cured (2 h at 300°C in an N_2_ environment). Interconnects' metallization layer of Ti/Au/Ti (6/225/6 nm) was sputtered on the polyimide substrate after O_2_ plasma surface activation. The conductive tracks were patterned by photolithography (2 μm width) and wet or dry etching of the gold (Au) and titanium (Ti), respectively. Next, a 3 μm‐thick encapsulation layer of polyimide was spin‐coated and baked as described previously. The 6 μm‐thick polyimide stack was next patterned using photolithography and O_2_ reactive ion etching to outline the implant and expose connection pads. To promote the adhesion between PI and the successive PDMS layer, a thin SiO_*x*_ layer (25 nm) was sputtered following O_2_ plasma activation. Next, a 35 μm‐thick layer of PDMS (10:1) was spin‐coated after an O_2_ plasma surface activation of the SiO_*x*_ and cured (2 h at 80°C). To integrate the μ‐LED bare dies (240 × 320 × 140 μm^3^, 470 nm) on the wafer, a Sn/Bi/Ag solder paste was first dispensed on the pads through stamping with a pick‐and‐place tool. Then, the μ‐LEDs were aligned and placed onto the solder paste using the same equipment, and a reflow was performed to ensure mechanical and electrical stability. Next, the μ‐LEDs were encapsulated with a 13% by‐weight ratio solution of polyisobutylene in cyclohexane to protect from water permeation. The light emission spectrum of the LED was modified by precisely dispensing a mixture of a 1:1 yellow phosphor and PDMS, cured at 75°C for 3 h. An additional drop of PDMS was dispensed and cured after O_2_ plasma surface activation. The pads were soldered to copper wires and then sealed with silicone. Finally, the μ‐LED array was released from the wafer by anodic dissolution of the Al film by applying a 1.5 V bias in saturated NaCl solution.

*Micro‐LED array characterization*. The emission spectrum of the yellow LEDs was measured with a spectrometer. The electrical performance of the optoelectronic implants (*n* = 5) was characterized by applying an increasing current to the 4 parallel μ‐LEDs and measuring the output voltage.

The total optical power emitted at 560 nm was measured with a photodiode and a power meter, and the power density was calculated at the relevant driving voltage (3.3 V).

Lastly, the thermal characterization of the μ‐LED array was performed by placing an unsheathed fine gage thermocouple (0.05 mm diameter, type K) on the encapsulated LEDs and measuring the temperature increment during LED operation (80% duty cycle, 1 Hz, 20 s duration) at various optical power densities. In vivo conditions were mimicked by placing the arrays in a 3% w/v agarose and PBS1x environment.

### 2.4 Surgeries

Surgical procedures were performed under general isoflurane anesthesia (2.0%, mixed with O_2_). Body temperature was maintained with a heated surgical pad, and eyes were protected with ophthalmic gel.

*Implant*. Under sterile conditions, skin incisions exposed the skull and the proximal part of the leg. Fasciae under the skin were detached from the muscles to create space for the implant and the wires. A straight glass pipette was used to pass the implant wires from the leg to the head.

Two holes were drilled in the skull, and precision screws were superficially positioned. The three branches male–male connector was positioned, and two wires of the implant were soldered to the connector. The wires were positioned around the screws, and the ensemble was solidarized and anchored with glue and dental acrylic.

The implant was carefully pulled below the sciatic nerve, and the light‐emitting μLED structures were softly placed around with parallel suture threads through the array's anchoring sites. The subcutaneous connector was attached to the biceps femoris muscle and carefully placed to reduce the constraints along the device. The hindlimb muscles were sewed back together, and the skin incision was closed with sutures, surgical glue, and clips.

For postoperative analgesia, animals received an injection of Carprofen (5 mg/kg). Mice received 500 μL of NaCl 0.9% intraperitoneally after the surgery for hydration and were placed in single‐housed cages with Paracetamol ad libitum in the water for 48 h.

*Spared nerve injury* (*SNI*). The SNI surgery was performed according to the original model.^37^, ^38^ The tibial and common peroneal branches of the sciatic nerve were ligated and sectioned, while the sural branch remained intact. Muscles and skin were then sutured. The sham surgery served as a control and followed the same procedure, placing a thread along the nerve but without nerve ligation and section.

### 2.5 Optogenetic stimulations

In vitro *stimulations*. Dissociated DRG neurons were stimulated in vitro by using a CoolLED pE‐340fura equipped with an LED eGFP pE‐300 filter set. The stimulation consisted of a 1 s continuous yellow light stimulation at 575 nm, 100% system amplitude intensity.

In vivo *stimulations*. In vivo light stimulation was performed through the optoelectronic implant during the whole behavioral testing session. A 3.3 V current was applied to illuminate the four μLEDs on the implant. The stimulation parameters were the following: 20s light train pulses (590 nm), 1 Hz frequency, 80% duty cycle (800 ms ON/200 ms OFF), and an inter‐train interval of 1 s.

### 2.6 Behavior

*Mechanical sensitivity*. Mechanical sensitivity and nociception were measured using calibrated Von Frey monofilaments applied on the lateral plantar hindpaw. First, the mechanical threshold toward non‐nociceptive mechanical stimulations was measured by applying a series of 6 monofilaments (0.008–2.0 g, starting with 0.16 g), using the up‐and‐down method.^39^, ^40^ Second, the number and percentage of responses toward nociceptive stimulation were assessed with a 4.0 g monofilament that was applied 10 times.

*Thermal sensitivity*. The lateral plantar hindpaw was exposed to a radiant heat beam.^41^ The latency for paw withdrawal was measured (cut‐off at 20 s). The heat stimulation was repeated 3 times for each paw, and the mean of latencies was calculated.

*Formalin test*. The formalin test was performed by ethological observations and recordings of the mice, after subcutaneous injection of 10 μL formalin (5%) in the hindpaw. The time spent lifting, shaking, and licking the paw was measured for 80 min (5 min bouts).

### 2.7 Immunohistochemistry and image analysis

Mice were euthanized with pentobarbital (50 mg/kg) and underwent intracardiac perfusion of NaCl (0.9%) followed by paraformaldehyde PFA (4%). Tissues were collected and post‐fixed, then cryoprotected in a sucrose solution (20%) overnight. Sections of 12 μm for nerve and DRGs, or 25 μm for spinal cord, were cut with a cryostat. Sections were incubated for 1 h at room temperature with 250 μL blocking solution (normal goat serum 10%, Triton 100× 0.3% and PBS 1×) then overnight at 4°C with the primary antibody (rabbit anti‐NF200 (1/200), rabbit anti‐CGRP (1/1000), rabbit anti‐Peripherin (1/2000)) or the biotinylated isolectin B4 (1/50). Anti‐rabbit antibody conjugated with Cy3 (1/400) was used as a secondary antibody while IB4 was revealed with Streptavidin conjugated with aminomethylcoumarin acetate AMCA (1/200).

Representative images were acquired on a confocal microscope (20×). Quantification was performed on epifluorescent images, obtained from an Axio Scan microscope, and analyzed with Fiji software. The quantification was performed on 3 mice from which L3 to L5 DRGs were used (4–6 sections/mouse/DRG). The measures were normalized over the surface of the soma's area within the DRG and expressed as a number per mm^2^. The average number of positive cells was calculated for each mouse.

*DRG neuron dissociation*. The ipsilateral and contralateral L3–L5 DRG of SNS‐Cre/ArchT‐eGFP and littermate mice were harvested and immediately placed in a cold HBSS solution. The DRG were enzymatically dissociated in Dispase II (1 mg/mL) and Collagenase A (2 mg/mL), prepared in HBSS, for 1 h at 37°C. Then, the DRGs were transferred to culture media composed of Neurobasal Plus Medium, supplemented with B27 Plus Supplement (2%) and Penicillin/Streptomycin (1%), and thus triturated with a fire‐polished glass pipette. DRG cells were seeded on poly‐D‐lysin‐coated glass coverslips and incubated at 37°C.

### 2.8 Electrophysiology: Whole‐cell patch clamp recordings

*The intracellular solution contained*: 140 mM KCL, 5 mM HEPES, 2 mM ATP disodium, 1 mM MgCl2, and 0.5 mM EGTA, adjusted to pH 7.3 with KOH, osmolarity 305 mosM. The extracellular solution contained: 140 mM NaCl, 10 mM HEPES, 3 mM KCl, 2 mM MgCl2, and 2 mM CaCl_2_, adjusted to pH 7.2 with NaOH, osmolarity 315mosM.

*Data recordings*. We performed whole‐cell recordings of small DRG neurons (diameter < 30 μm).^42^ We used the amplifier MultiClamp 700B, Digidata 1440A series, and pClamp 10.7 software for data acquisition. The cells were visualized under a microscope and stimulated by using a CoolLED pE‐340fura equipped with an LED eGFP filter set and a digital camera ORCAFlash2.8 under the control of the Olympus CellSens v3.2 acquisition software. The ET560/40× filter with a transmission of yellow light from 540 to 580 mm was used for optogenetic inhibition. Borosilicate patch pipettes had a resistance of 2–4 MΩ—built with a microforge MF200‐2, H4 platinum/iridium wire.

Cells were sealed and recorded in current‐clamp mode. We used data only from cells with a tight Giga seal and stable recordings, in voltage‐clamped at −60 mV, and maintained a stable seal during the recording assessed by the input resistance during‐100 pA hyperpolarization step for 200 ms. The protocol lasted 4 s per step and included two pulses of 5 ms increasing by 10 pA after each step until 500 pA, with the second pulse delivered in the middle of a 1 s yellow light stimulation. Images of the patched cells were acquired in bright field and epifluorescence to measure the diameter and intensity of eGFP fluorescence. The resting membrane potential, the membrane potential during yellow light exposure, and the rheobase, with and without yellow light, were measured using Clampfit 10.7 software.

### 2.9 AAV vector production

Serotype 6 AAV vector (AAV‐cmv‐ArchT) was produced by transient transfection of adherent HEK293‐AAV using calcium phosphate precipitation. Six hours after transfection, the cell culture medium was supplemented with 4 mM valproic acid. The cells were collected 72 h after transfection and were lysed by repeated three‐thaw cycling. AAV6 particles were purified on an iodixanol gradient followed by heparin binding chromatography. The titer was determined by qPCR using an amplicon located in the beta‐globin intronic sequence.

### 2.10 Cells preparation, infection and electrophysiology recordings

DRG neurons from control littermates were harvested, dissociated as described in the main method, and left to seed in the incubator at 37°C for 3 h in 1 mL of DRG media. Then, 2 μL of an AAV6‐CMV‐ArchT‐GFP (titer of 2.27 × 10^13^ vg/mL) was added to the culture medium and incubated overnight at 37°C. The GFP^+^ cells were recorded by whole‐cell patch clamp recordings, following the same experimental procedure and protocol as described above. Statistical analyses were performed accordingly.

### 2.11 Statistical analyses

The statistical analyses are described for the different datasets in the Supplemental Data Table 2 Statistical Analysis. These analyses were performed using GraphPad Prism and/or R software.

## 3 RESULTS

### 3.1 Physical properties of optoelectronic implant for sciatic nerve

**FIGURE 1:**
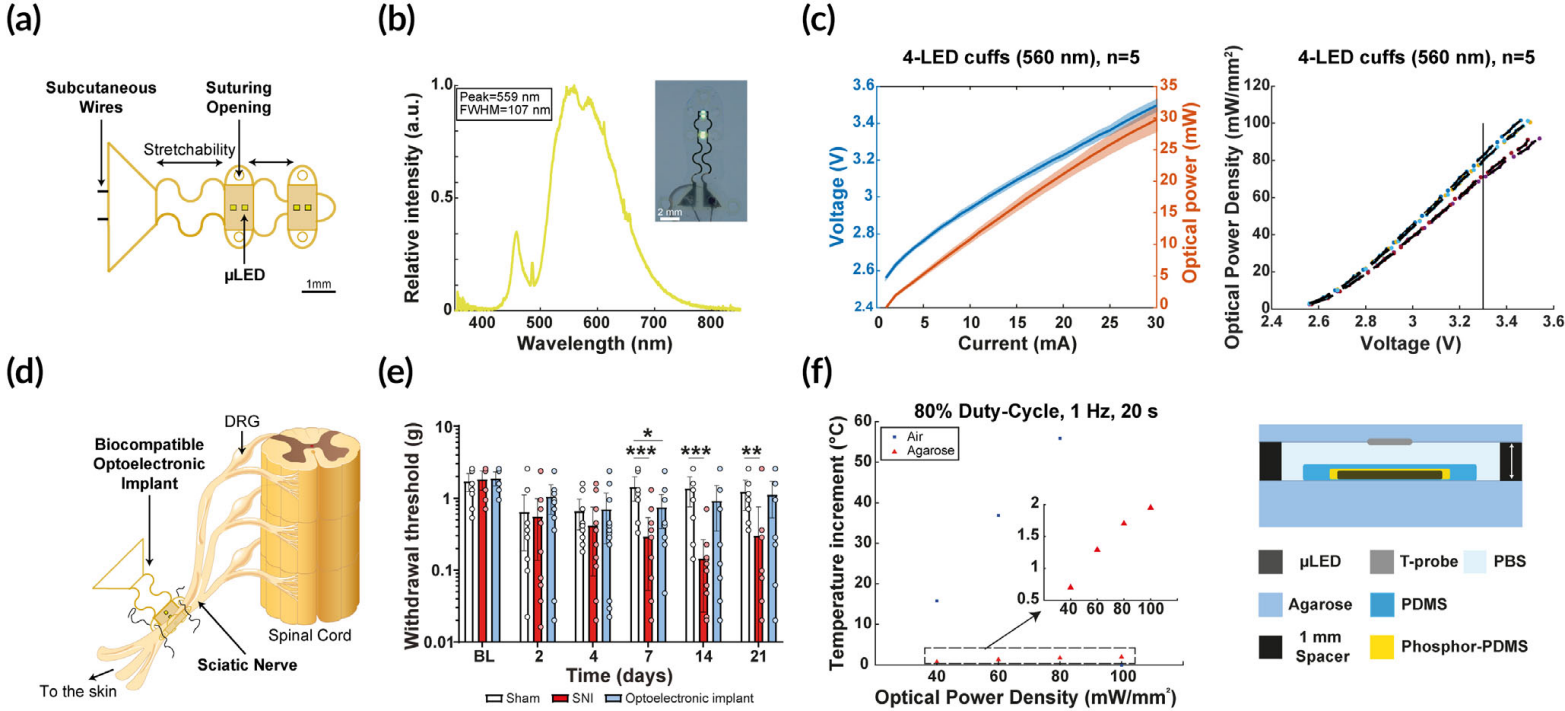
Optoelectronic biocompatible implant. (a) Schematic drawing of an implant including the stretchable gold interconnects as well as the four *μ*‐LEDs simultaneously activable. (b) Light spectrum of the phosphor‐loaded silicone LED for specific yellow light output. Inset: Picture of fabricated implant shining yellow light. (c) Physical properties of the optoelectronic device. On the left, optoelectrical performances of yellow light implants in relation to the applied current. On the right, optical power density in relation to the applied voltage. The in vivo experiment with the implants applied a 3.3 V corresponding to a 60–80 mW/mm^2^ optical power density. (d) Anatomical drawing of an implant positioned around the sciatic nerve. (e) Mechanical sensitivity measured by the Von Frey test from 2 to 21 days after implantation or spared nerve injury (SNI) surgeries. Implanted animals (blue) are compared to sham (white) and SNI (red) operated animals. (*N* = 12–14. Two‐way ANOVA with Tukey's multiple comparisons, *p < 0.05; **p < 0.01; ***p < 0.001 in comparison with the sham group). (f) Temperature changes upon light stimulation parameters equivalent to the vivo experiments (20 s ON, 80% Duty‐Cycle, 1 Hz frequency, 60–80 mW/mm^2^). These measures were performed in an air or agarose environment (mimicking tissue conditions), as shown in the schematic representation of the experimental setup (right scheme) for temperature increment assessment.

We have fabricated soft optoelectronic implants to deliver yellow or blue light to the sciatic nerve of mice.^23^, ^32^ The implant can be controlled either by a miniaturized wireless electronic interface or with standard wires and instrumentation. The implant integrates four μ‐LEDs of 320 μm × 240‐μm surface area on the polyimide substrate. Serpentine gold interconnects patterned on polyimide ensure stretchability and reliable electromechanical performance by accommodating movements of the nerve (Figure 1a). The entire system is then further encapsulated in soft PDMS silicone. By dispensing a phosphor‐loaded silicone over the LED (Figure 1b) it is possible to convert the emitted wavelength from 470 to 560 nm to match the ArchT response spectrum (559 nm).

Devices prepared for implantation showed reliable and reproducible opto‐electrical performance (Figure 1c). For the selected stimulation parameters (3.3 V) the applied optical power density corresponded to a range of 60–80 mW/mm^2^ (Figure 1c) which is a sufficiently high value to guarantee opsin response even in the event of light diffusion and scattering around the surrounding tissue. Monitoring of temperature for the selected stimulation paradigm applied in vivo (80% DC, 1 Hz, 60–80 mW/mm^2^) was carried out on the bench in air as well as in a PBS and agarose 3% weight/volume environment (Figure 1f). The agarose and PBS preparation simulated the effects of surrounding neural tissue, muscle, and fat (thermal conductivity of tissue: 0.2–0.6 W/mK,^43^ agarose: 0.5–0.6 W/mK,^44^ PBS: 0.5–0.6 W/mK^45^, ^46^). A limited temperature increase in agarose (below 2°C) after 20 s stimulation was measured locally above the first LED site, whereas in air devices failed when reaching operating temperatures >80°C. Mechanical stability and LED encapsulation efficacy have been previously demonstrated through cyclic stretching at 20% strain and through accelerated aging tests in saline at 67°C, respectively.^32^

The innocuity of in vivo biointegration of the optoelectronic implant on sensory properties of the sciatic nerve (Figure 1d) was assessed by testing mechanical threshold response after low‐intensity mechanical stimulation of the skin territory of the nerve. If the nerve is severed by the implantation, a lower threshold toward innocuous stimulus would develop, suggesting neuropathic pain‐related behavior. Here, wild‐type mice were implanted with non‐functional prototypes. Seven days after surgery, the mechanical sensitivity of implanted mice was comparable to the sensitivity of the sham group, as opposed to the SNI group that developed mechanical allodynia‐like behavior (^38^) (time post‐injury effect, *p* < 0.0001, *F* (5, 175) = 13.8; surgery effect, *p* = 0.001, *F* (2, 35) = 8.47; interaction effect, *p* = 0.015, *F* (10, 175) = 2.29; *n* = 12–14 mice/group) (Figure 1e). Post hoc analysis revealed that after SNI, mice displayed at any time point a significantly lower mechanical threshold than at baseline, indicating that they exhibit a mechanical allodynia phenotype.

### 3.2 Distribution of ArchT

**FIGURE 2:**
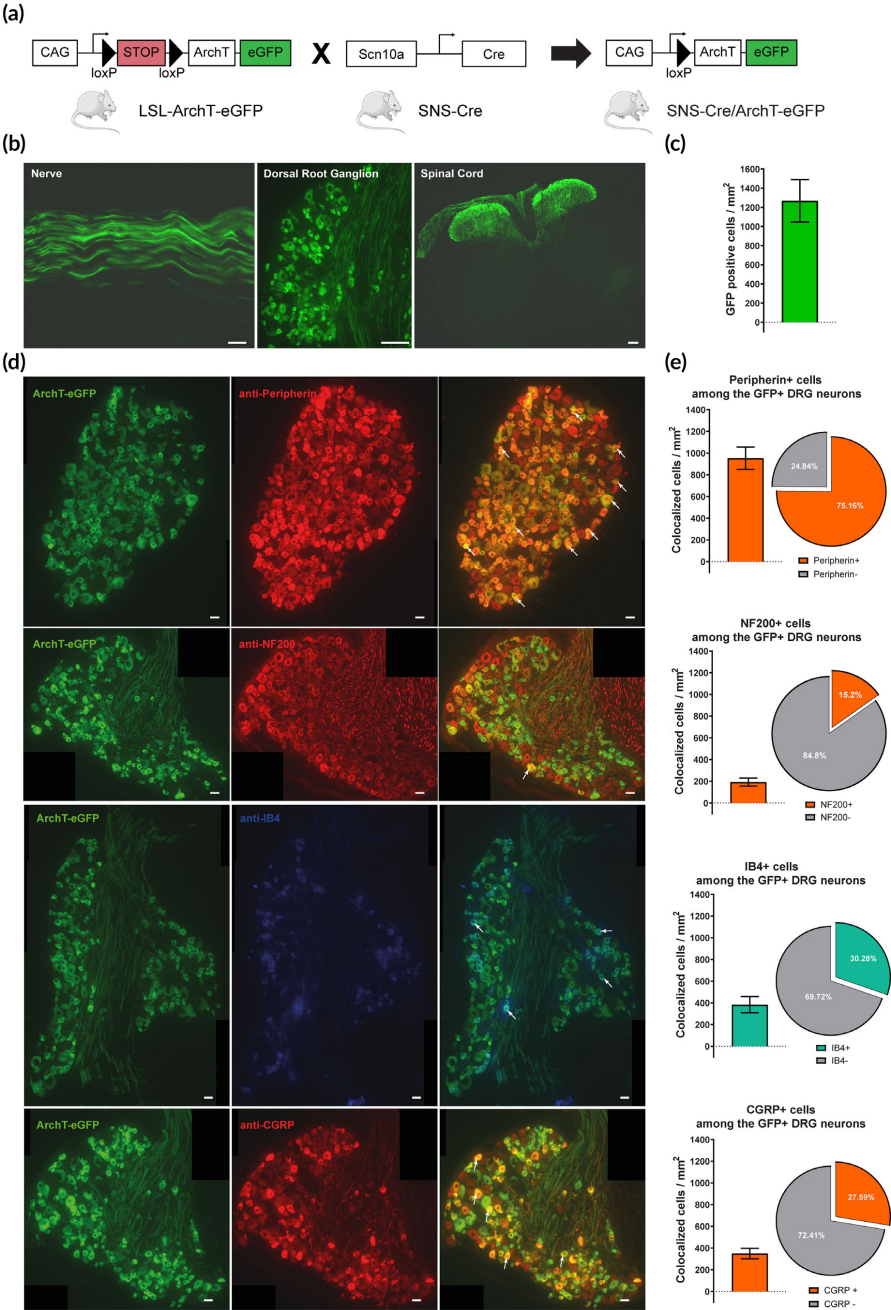
Distribution of ArchT expression in the DRG of SNS‐ArchT mice. (a) Schematic representation of the transgenic mouse line construction. Scn10a gene encodes for the protein of the tetrodotoxin‐resistant voltage‐gated sodium channel alpha subunit Na_v_1.8; LSL encodes for the STOP sequence flanked with two loxP sites. (b) Representative images of ArchT‐eGFP expression into the sciatic nerve, dorsal root ganglion, and spinal cord sections. Scale bar = 200 μm. (c) The quantification of GFP‐positive neurons into the DRG indicates an average of 1267.9 ± 288.7 GFP+ cells/mm^2^. *N* = 58–69 sections counted per animal and obtained from 3 mice. (d) Representative images of the expression of ArchT‐eGFP with peripherin (small nociceptive DRG neurons), NF200 (large DRG neurons), IB4 (non‐peptidergic small DRG neurons), or CGRP neurons (peptidergic small DRG neurons). Arrows indicate positively colocalized cells. Scale bar = 50 μm. (e) Quantification of GFP+ cells which are Peripherin^+^ (952.9 ± 103.5), NF200^+^ (192.7 ± 36.1), IB4^+^ (383.9 ± 74.0) or CGRP^+^ (349.8 ± 48.5). Additionally, the proportion of Peripherin^+^ (75.16 ± 8.16%), NF200^+^ (15.2 ± 2.85%), IB4^+^ (30.28% ± 5.84%) or CGRP^+^ (27.5% ± 3.82%) neurons, among the ArchT‐eGFP positive neurons, are also represented. Scale bar = 50 μm. For each staining, *n* = 14–19 sections per animal were counted and obtained from 3 mice.

We constructed a transgenic mouse line that permits optogenetic inhibition of nociceptive neurons. The SNS‐ArchT‐GFP mouse line expresses the inhibitory archaerhodopsin (ArchT) in Na_v_1.8 (also termed SNS) expressing neurons (Figure 2a). The histological analysis demonstrates the substantial distribution of the opsin in the nerve, primary sensory neurons somas, and spinal cord central terminals of DRG neurons (Figure 2b). The quantification of GFP^+^ cells within the DRG showed a density of 1267.9 ± 288.7 GFP^+^ cells/mm2 (Figure 2c). Co‐staining with other markers revealed that 75.16% ± 8.16% of GFP^+^ neurons are peripherin^+^ and 15.2% ± 2.85% are NF200^+^ (for small nociceptive or larger DRG neurons, respectively^47^), indicating a preferential expression of ArchT in small nociceptive neurons (Figure 2d, e). Moreover, among the ArchT‐eGFP^+^ neurons, 27.59% ± 3.82% are CGRP^+^, and 30.28% ± 5.84% are IB4^+^, which shows that peptidergic and non‐peptidergic nociceptive neurons are similarly affected (Figure 2d, e).

### 3.3 Functional validity of ArchT in the transgenic mouse line

**FIGURE 3:**
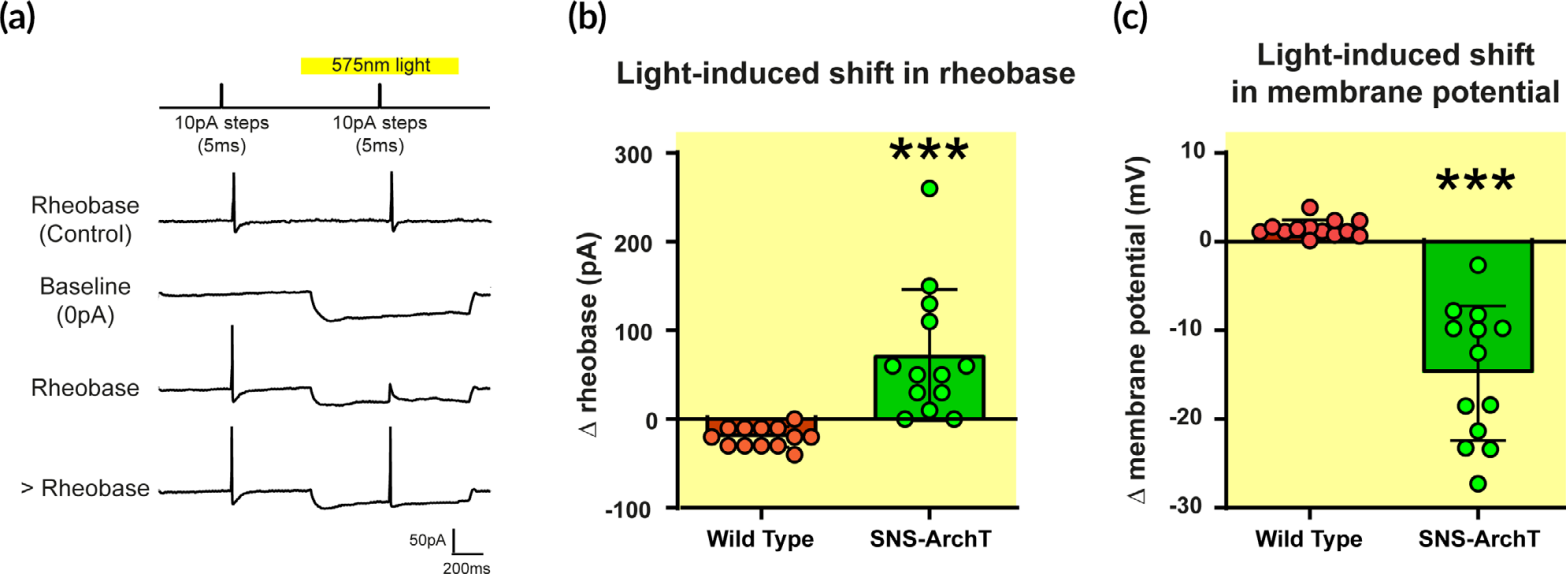
Optogenetic inhibition of dissociated DRG neurons expressing ArchT. (a) Representative traces of recordings in the different conditions. The current clamp protocol consists of two depolarising 5 ms steps increased by 10pA after every sweep to determine the rheobase, without and then with yellow inhibitory light (575 nm). (b), (c) Quantified effect of optogenetic inhibition on (b) the rheobase (*N* = 13 cells/group. Unpaired *t*‐test with welch correction, *t*(12.59) = 4.46, ***p < 0.001) and (c) the membrane potential (*N* = 13 cells/group. Unpaired *t*‐test with welch correction, *t*(12.38) = 7.696, ***p < 0.001) in DRG neurons of SNS‐Cre ArchT‐GFP (green) and wild type control littermates (red).

The functional effect of light‐induced neuronal inhibition is confirmed by in vitro patch clamp recordings of small SNS‐ArchT‐GFP+ neurons in dissociated primary culture of lumbar DRGs. The current clamp protocol delivered two 5 ms pulses 1 s apart, in 10 pA increasing steps, one with and one without yellow light (Figure 3a). The electrophysiological properties of DRG neurons of transgenic SNS‐ArchT‐GFP mice and control littermates are similar in the absence of yellow light stimulations (Supplemental Figure S2A–F). Yellow light decreased the excitability of GFP+ neurons expressing the inhibitory opsin: upon yellow light stimulation, a higher current amplitude was required to elicit an action potential, defined as the rheobase (Figure 3b) (control littermates vs. SNS‐ArchT, *p* = 0.0007, *t*(12.59) = 4.46, *n* = 13 cells/genotype). The light‐induced hyperpolarization of the resting membrane potential (Figure 3c) (control littermates vs. SNS‐ArchT, *p* < 0.0001, *t*(12.38) = 7.696, *n* = 13 cells/genotype). These data confirm the functionality of the ArchT in GFP+ neurons and the ability of yellow light to inhibit the small primary sensory neurons in the SNS‐ArchT‐GFP transgenic mouse line.

### 3.4 AAV‐driven functional ArchT in vitro the transgenic mouse line

The delivery of a functional ArchT opsin has been assessed by in vitro patch clamp recordings of dissociated DRG primary neurons, transduced with AAV6‐CMV‐ArchT‐eGFP (Representative traces shown in Figure S3A). The functionality of the inhibitory opsins expressed by the AAV6 vector was confirmed by a significant increase in the rheobase (Figure S3B) (control GFP^−^ vs. transduced GFP^+^ cells, *p* < 0.0001, *t*(19.29) = 5.687, *n* = 10–16 cells/group), together with a decrease in the membrane potential observed upon yellow light stimulus (Figure S3C) (control GFP^−^ vs. transduced GFP^+^ cells, *p* < 0.0001, *t*(19.2) = 6.454, *n* = 9–16 cells/group), in comparison with the non‐transduced neurons. These data confirm the use of AAV6‐CMV to express a functional ArchT channel in DRG neurons and the ability of yellow light to inhibit them. The transduction with the virus is a proof of concept for gene therapy to be used in combination with optoelectronic implants to inhibit chronic pain.

### 3.5 Behavioral recordings in the transgenic mouse line

**FIGURE 4:**
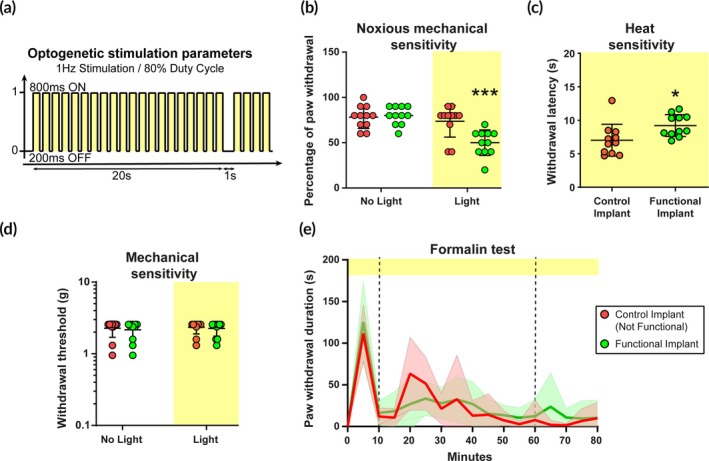
Effect of yellow light inhibition on the behavioral response after innocuous and nociceptive (painful) stimuli. (a) Optogenetic stimulation parameters: 20 s light train pulses (590 nm), 1 Hz frequency, 80% duty‐cycle (800 ms ON/200 ms OFF) and an inter‐train interval of 1 s. (b) Response to nociceptive mechanical stimuli (4 g. Von Frey filament) is reduced when the optoelectronic implant is on (*N* = 11/group. ANOVA two‐way repeated measures, with Sidak's multiple comparisons, ***p < 0.001 in comparison with the control group). (c) The latency of paw withdrawal is increased when the optoelectronic implant is on after thermal stimuli tested on the (Hargreaves test) (*N* = 11/group. Unpaired *t*‐test with welch correction, *p = 0.022). (d) Response to innocuous mechanical stimuli is unchanged when the optoelectronic implant is on (Von Frey up and down method, functional (green) or control conditions (red) (*N* = 11/group. ANOVA two‐way repeated measures, no significant effect of the Stimulation factor *p* = 0.7321; the Group factor *p* = 0.7672; nor Interactions *p* = 0.2131). (d) Measurement of paw withdrawal duration (in s) after intraplantar formalin injection on the left implanted paw. (*N* = 9–10/group. ANOVA two‐way repeated measures, Time factor effect *p* < 0.0001; no significant effect of the group factor *p* = 0.5733; nor interactions *p* = 0.2393).

The effect of yellow light on pain sensitivity was recorded in vivo in SNS‐ArchT transgenic mice implanted with functional optoelectronic devices. A protocol of 80% Duty‐Cycle, 1 Hz, 60–80 mW/mm^2^ (Figure 4a) was applied and its effects were evaluated during four types of stimuli (*n* = 11 mice/group) (experimental design in Supplemental Figure S4A). Withdrawal behavior was recorded upon non‐nociceptive (innocuous) mechanical stimulus with Von Frey monofilaments (up‐and‐down method) and mechanical nociceptive stimulus (mild painful, with the application of a strong Von Frey monofilament of 4 g) as well as nociceptive heat stimulus (Hargreaves test). In addition, spontaneous behavior was recorded during acute and intense nociceptive inflammatory stimulus evoked by the injection of Formalin (3%) in the paw. The control group is composed of SNS‐ArchT‐eGFP mice implanted with a non‐functional device and control littermates implanted with a functional device.

The light applied through the optogenetic implant induced a significant reduction of the paw withdrawal responses toward painful stimuli, evoked either with the 4 g monofilament for mechanical modality (stimulation effect, *p* < 0.0001, *F* (1, 20) = 26.94; group effect, *p* = 0.036, *F* (1, 20) = 5.053; interaction effect, *p* = 0.0011, *F* (1, 20) = 14.63; *n* = 11 mice/group) (Figure 4b), or through a radiant heat beam for the thermal sensitivity (*p* = 0.0219, *t*(17.68) = 2.513, *n* = 11 mice/group) (Figure 4c). Conversely, no significant differences in the thermal sensitivity were observed between both groups on the right non‐implanted paw (*p* = 0.6358, *t*(15.13) = 0.4833, *n* = 11 mice/group) (Supplemental Figure S4D). Importantly, low‐threshold mechanical sensitivity measured with the Von Frey, standard up‐and‐down method (0.16–2 g), revealed no significant differences between groups, both with and without light, on both paws suggesting the lack of significant somatosensory deficit induced by the optogenetic inhibition (Left implanted paw, stimulation effect, *p* = 0.7321, *F* (2, 40) = 0.3142; Group effect, *p* = 0.7672, *F* (2, 40) = 0.09; Interaction effect, *p* = 0.2131, *F* (2, 40) = 1.607; Right non‐implanted paw, stimulation effect, *p* = 0.794, *F* (2, 40) = 0.2319; group effect, *p* = 0.3188, *F* (1, 40) = 1.045; interaction effect, *p* = 0.8437, *F* (2, 40) = 0.1707; *n* = 11 mice/group) (Figure 4d and Supplemental Figure S4B). To test high intensity stimulation and inflammatory‐induced sensitization, animals were injected with formalin in the hind paw 12 days post‐implantation. The optogenetic stimulation via the sciatic implant emerged as being inefficient to reduce the formalin‐induced pain‐like behavior (time post‐injection effect, *p* < 0.0001, *F* (5.16, 87.72) = 27.37; Group effect, *p* = 0.5733, *F* (1, 17) = 0.3298; interaction effect, *p* = 0.2393, F(16, 272) = 1.237; *n* = 9–10 mice/group) (Figure 4e), both in the initial acute phase (*p* = 0.4405, *t*(15.09) = 0.7923, *n* = 9–10 mice/group) and subsequent sensitization phase (*p* = 0.1144, *t*(16.98) = 0.9102, *n* = 9–10 mice/group) (Supplemental Figure S4C).

## 4 DISCUSSION

Here, we used a miniaturized LED implant wrapped around the sciatic nerve to promote neuronal inhibition, leveraging optogenetic light stimulation and inhibitory opsin ArchT. To our knowledge, this represents the first description of a sciatic optoelectronic implant used for optogenetic inhibition of primary sensory neurons. Our results indicate that the 4 μLED on the implant, driven at 3.3 V, emits light in the expected range (Peak at 559 nm) and at an optical power relevant for ArchT opsins stimulation.^29^

The implantation around the sciatic nerve in mice is technically challenging, with the risk of a nerve injury and the potential constraints on the nerve during movements. The sciatic nerve implant exhibits strong biocompatibility through materials like polyimide (PI), titanium (Ti), gold (Au), PDMS, and SiO_*x*_, commonly used in biomedical applications. Our behavioral data showed that the implanted animals express a comparable mechanical sensitivity level to Sham‐operated mice, suggesting that the biocompatible optoelectronic implant is well‐tolerated and reliable. The ethological observation of the implanted animals in their home cage revealed no signs of discomfort or motor deficits. The heat produced by the light‐emitting implant remains in a moderate range when placed in conditions mimicking the in vivo situation. The soft optoelectronic implant demonstrates promising biocompatibility and safety, supported by mechanical stability, thermal performance, and neural integration assessments. Cyclic stretching (20% strain) and accelerated aging in saline (67°C) confirmed durability, while temperature monitoring showed only a 2°C increase in agarose, suggesting minimal heat‐related risks. Nonetheless, thermal tests were conducted ex vivo, and potential in vivo long‐term effects remain to be assessed. Further histological and functional experiments would be required to fully ensure the reliability, effectiveness, and long‐term safety of our implant, over weeks and months.

Previous descriptions of similar sciatic implants have proven not to induce any type of injury and/or inflammatory reaction.^23^, ^32^ The optogenetic excitation of TRPV1‐ChR2^+^ neurons, through a comparable sciatic optoelectronic implant, was shown to activate the immune system and further amplify the immune reaction induced by CFA intraplantar injections.^32^ Thus, it would be of particular interest to investigate whether nociceptor inhibition would trigger the immune system differently, leading to a beneficial effect for the relief of a pathological condition.

In the present study, the optoelectronic implant is used on SNS‐ArchT‐eGFP transgenic mice. As previously described,^36^ the histological analysis reveals an expression of the ArchT in the skin, the peripheral nerve, preferentially in the soma of the nociceptor neurons and in the dorsal horn of the spinal cord. The distribution of ArchT‐expressing neurons among IB4^+^, CGRP^+^, and Peripherin^+^ classes is consistent with known proportions of nociceptive neurons, reinforcing the specificity of our optogenetic approach. However, while NF200 and Peripherin provide valuable insights into fiber classification, they also have limitations. NF200 can co‐express with CGRP or SP and is not exclusive to *Aβ* fibers, as it is also present in a few *Aδ* fibers. Similarly, Peripherin, despite its association with C fibers, does not strictly label nociceptive neurons, as a few C fibers are non‐nociceptive. Given these constraints, a more refined characterization approach (incorporating immunohistochemistry, intracellular recordings, and transcriptomic analysis) would enhance specificity. Nevertheless, the well‐characterized SNS‐Cre line allows us to consider that the SNS‐ArchT line is not strictly limited to nociceptors, but highly enriched in this population.

Electrophysiological recordings of DRG neuron activity confirmed that the optogenetic inhibition through ArchT opsin reduces their excitability by hyperpolarizing the resting membrane potential. Similarly, the yellow light‐induced neuronal activity inhibition was confirmed on wild‐type DRG neurons transduced in vitro with an AAV‐expressing ArchT channel. ArchT activation allows for decreased neuronal excitability by hyperpolarizing the neuronal membrane and therefore increasing the rheobase.^18^, ^29^ Interestingly, an injection of a strong current remains capable of eliciting an action potential concomitantly with ArchT activation.^29^

Our behavioral recordings demonstrate that by targeting Na_v_1.8 expression in nociceptive neurons, we can selectively dull the mechanical and thermal acute nociception using optogenetic tools. Remarkably, the selective inhibition of Nav1.8‐expressing neurons did not influence low‐threshold mechanical somatosensation, despite a non‐negligible expression of ArchT in larger primary sensory neurons. The selective inhibition of nociceptive pathways can be concluded from the absence of changes in responses to low‐threshold mechanical stimuli. Nevertheless, further experiments would be required to fully rule out that other behavioral functions (such as locomotor activity, muscular strength, motor coordination…) are not impacted.

The formalin model provokes an acute and intense chemical stimulation of the paw and subsequent inflammatory sensitization that could not be optogenetically inhibited with the sciatic implant. Transdermal optogenetic inhibition of Na_v_1.8^+^ neurons has previously been shown to promote a modest behavioral relief of the hypersensitivity induced with other inflammatory compounds, such as CFA or zymosan.^18^ Such discrepancies could come from the different inflammatory agents that have been shown to exhibit dissimilar pharmacological properties leading to different cellular and behavioral effects.^48^

Although ArchT activation is efficient in silencing synaptic transmission, it is also accompanied by transients' pH fluctuations, which have been suggested to be necessary for the inhibitory effect of the opsin, at least in hippocampal circuits.^49^ This work proposed that optogenetic inhibition by ArchT channels does not prevent the transmission of action potentials along the nerve. It would represent a limitation for the use of optoelectronic implants positioned around the sciatic nerve, in between the peripheral cutaneous territory and the spinal cord. Despite a reduction of neuronal excitability which is sufficient to reduce the behavioral response toward a nociceptive stimulation, it would be insufficient to prevent responses to higher intensity stimulations such as with the formalin cutaneous injection.

The efficiency of peripheral optogenetic inhibition also lies in the pattern of stimulation. Indeed, various neuronal activity patterns contribute to different gene regulation^50^ and may constitute an essential characteristic for the peripheral sensitization mechanisms. The pattern of peripheral neuron activity differs from different stimulus types^51^ and the inability of our optoelectronic sciatic implant to elicit a significant inhibition of the inflammatory pain after formalin could be related to the pattern of ArchT activation.

As previously proposed for bladder contraction control,^28^ it would be highly relevant to integrate a recording system into the device, allowing one to selectively modulate the neuronal activity only when it exceeds a certain threshold.

The transdermal Na_v_1.8^+^ neuron inhibition previously showed a significant and large effect in the nerve injury model.^18^ The efficiency of the optoelectronic implant to inhibit abnormal discharges and pain hypersensitivity induced by nerve injury is a further step to investigate. In addition, a comparative study evaluating the behavioral pain responses with the optogenetics implant versus a standard pharmacological analgesic would be of interest in the future.

Taken together, our results advocate that reducing excitability of primary sensory neurons in vivo does not affect the transmission and perception of a non‐painful stimulus. It significantly reduces the behavioral response toward noxious stimuli but does not alter the perception of an intense stimulus. This optogenetic inhibition approach provides an advantageous gradation effect: it maintains innocuous somatosensory sensation intact and reduces the perception of moderate painful stimuli without affecting the necessary protective role of pain signaling when exposed to high‐intensity and harmful stimulation such as chemical exposure.

The translation of these optoelectronic implants could require thorough assessment and optimization of their durability, as well as their compatibility, to allow for long‐term benefits for patients. It will be important to assess any long‐term positive benefits, such as prevention or reversal of central sensitization, but also potential negative effects such as the development of resistance and dependence on optogenetic treatment. Unlike previous optogenetic pain management approaches requiring external fiber optics, our implant is fully biocompatible and provides precise, localized, wireless neuronal modulation with light. Its miniaturized design allows stable long‐term integration with minimal impact on nerve function, enhancing its translational potential. Beyond experimental applications, biocompatible optoelectronic implants have potential translational uses in combination with gene therapy technologies for the delivery of opsin transgene. By selecting appropriate viral vectors and gene promoters to deliver the transgene for the inhibitory opsin, gene therapy could allow to selectively inhibit a subpopulation of primary sensory neurons that become hyperexcitable in chronic pain conditions.^16^ This neuronal subpopulation selectivity would mitigate off‐target side effects. However, such gene therapy strategies need to ensure that the selected viral vector does not induce neuroinflammation, a process known to increase pain sensitivity, or any latent toxicity.^52^, ^53^

Importantly, considering sex/gender as a biological and sociocultural variable is essential for understanding pain mechanisms and developing effective, personalized interventions in both clinical and research settings. Although our study has not been designed to study the sex differences, the inhibitory effects of ArchT‐mediated optogenetics on nociceptive neurons seem robust across sexes. To ensure a generalizable translation of such implants, future studies will be required to explore sex‐specific responses to optoelectronic implants further.

## FUNDING INFORMATION

Bertarelli Foundation Catalyst Fund.

## CONFLICT OF INTEREST STATEMENT

The authors report no conflict of interest.

## Supporting information


**Supplemental Figure S1.** Simplified microfabrication process flow. (A) A 4″ Si wafer with a Ti/Al (10/100 nm) sacrificial layer is used as a carrier. (B) Spin‐coating and baking of a bottom 3‐μm layer of PI. (C) Sputtering and etching of Ti/Au/Ti interconnects (wet etching and RIE). (D) Spin‐coating and baking of a top 3‐μm layer of PI. (E) Patterning of PI outline and opening contact sites through O_2_ RIE. (F) Sputtering of 25 nm SiO_*x*_ adhesion layer and coating of 35 μm PDMS. (G) SiOx and PDMS RIE etching to pattern outline and open contact sites. (H) Solder paste stamping and μLED placement. (I) Encapsulation of μLED with PIB, yellow phosphor‐PDMS and PDMS, copper wire soldering, and RTV encapsulation. (J) Anodic Al dissolution for structure release from the wafer.

**Supplemental Figure S2.** Patch clamp recording of dissociated DRG neurons expressing ArchT. (A) Quantification of the fluorescence of the recorded neurons from SNS‐ArchT (green) or control (red) littermate mice (*N* = 12–13 cells/group. Unpaired *t*‐test with welch correction, *t*(19.76) = 20.62, *** *p* < 0.0001). The lower the optical density is the more fluorescent the cells are. (B) Quantified capacitance (in pF) of the recorded dissociated DRG neurons from SNS‐ArchT (green) or control (red) littermate mice (*N* = 12–13 cells/group. Unpaired *t*‐test with welch correction, *t*(22.86) = 2.009, *p* = 0.0565). The lower the capacitance is the smaller the recorded cells are considered. (C) Quantified cell diameter of the recorded dissociated DRG neurons from SNS‐ArchT (green) or control (red) littermate mice (*N* = 12–13 cells/group. Unpaired *t*‐test with welch correction, *t*(22.09) = 2.335, * *p* = 0.029). The difference is probably due to the selection of cells: fluorescent ones belong to a majority of small DRG neurons, while in control littermates the selection is random (no fluorescence). (D) Quantified input resistance of the recorded dissociated DRG neurons from SNS‐ArchT (green) or control (red) littermate mice (*N* = 12–13 cells/group. Unpaired *t*‐test with Welch correction, *t*(22.39) = 0.4298, *p* = 0.6713). (E) Quantified rheobase of the recorded dissociated DRG neurons from SNS‐ArchT (green) or control (red) littermate mice (*N* = 12–13 cells/group. Unpaired *t*‐test with welch correction, *t*(21.05) = 0.6215, *p* = 0.541). (F) Quantified resting membrane potential (RMP, in mV) of the recorded dissociated DRG neurons from SNS‐ArchT (green) or control (red) littermate mice (*N* = 12–13 cells/group. Unpaired t‐test with welch correction, *t*(22.48) = 2.159, * *p* = 0.0418).

**Supplemental Figure S3.** Optogenetic inhibition of dissociated DRG neurons in vitro transduced with an AAV6‐CMV‐ArchT‐GFP vector. (A) Representative traces of recordings in the different conditions. Current clamp protocol consists of two depolarising 5 ms steps increased by 10 pA after every sweep to determine the rheobase, without and then with yellow inhibitory light (575 nm). (B), (C) Quantified effects of optogenetic inhibition on (B) the rheobase (*N* = 10–16 cells/group; unpaired t‐test with welch correction, *t*(19.29) = 5.687, *** *p* < 0.001) and (C) the membrane potential (*N* = 9–16 cells/group; unpaired *t*‐test with welch correction, *t*(19.2) = 6.454, *** *p* < 0.001) in GFP^+^ DRG neurons (green), using the GFP^−^ DRG neurons as control (red).

**Supplemental Figure S4.** Optogenetic stimulation protocol and experimental design. (A) Experimental Design. Following the implantation, the animals went through a week of recovery and were tested for mechanical and thermal sensitivity without light first, then with light on (yellow lines). Then, they received an intraplantar injection of formalin 3%, and spontaneous paw withdrawal was measured for 80 min with yellow light stimulation (same stimulation parameters as previously: 20 s light train pulses, 1 Hz, 80% duty‐cycle, 1 s inter‐train interval). The control group were either transgenic mice implanted with non‐functional implants, or control littermates implanted with a functional implant. Both groups behaved similarly and were therefore pooled. (B) Mechanical sensitivity measured, using the standard Von Frey up and down method, on the right non‐implanted paw from functional (green) or control conditions (red) (*N* = 11/group. ANOVA two‐way repeated measures, no significant effect of the Stimulation factor *p* = 0.794, the Group factor *p* = 0.3188 nor Interactions *p* = 0.8437). (C) Measurement of the total paw withdrawal duration (in s), during the acute phase (0–10 min), or the latest phase (10–60 min), after intraplantar formalin injection (*N* = 9‐10/group. Unpaired *t*‐test with welch correction, no significant differences: (0–10 min) *p* = 0.3562; (10–60 min) *p* > 0.99). (D) Thermal sensitivity toward noxious stimulation, tested on the Hargreaves test, on the right non‐implanted paw (*N* = 11/group. Unpaired *t*‐test with welch correction, non‐significant differences *p* = 0.6358).

**Supplemental Data Table 1.**RRID and source references of the resources used in the current study.

**Supplemental Data Table 2.** Statistical table. Each row represents a dataset and the corresponding statistical tests, statistical values, *p*‐values, and effect size factors (generalized eta square *η* ^2^ or Cohen's *d*).

## Data Availability

The data that support the findings of this study are openly available (CC‐BY Rights) in Zenodo at https://doi.org/10.5281/zenodo.13757011, reference number 10.5281/zenodo.13757011
