# Supplementary figures and images for: Optogenetic modulation of peripheral nociceptive neurons with biocompatible optoelectronic implants
Source: Bioeng Transl Med. 2025 Jun 26;10(4):e70034. doi: 10.1002/btm2.70034 (PMC12284439; doi:10.1002/btm2.70034)

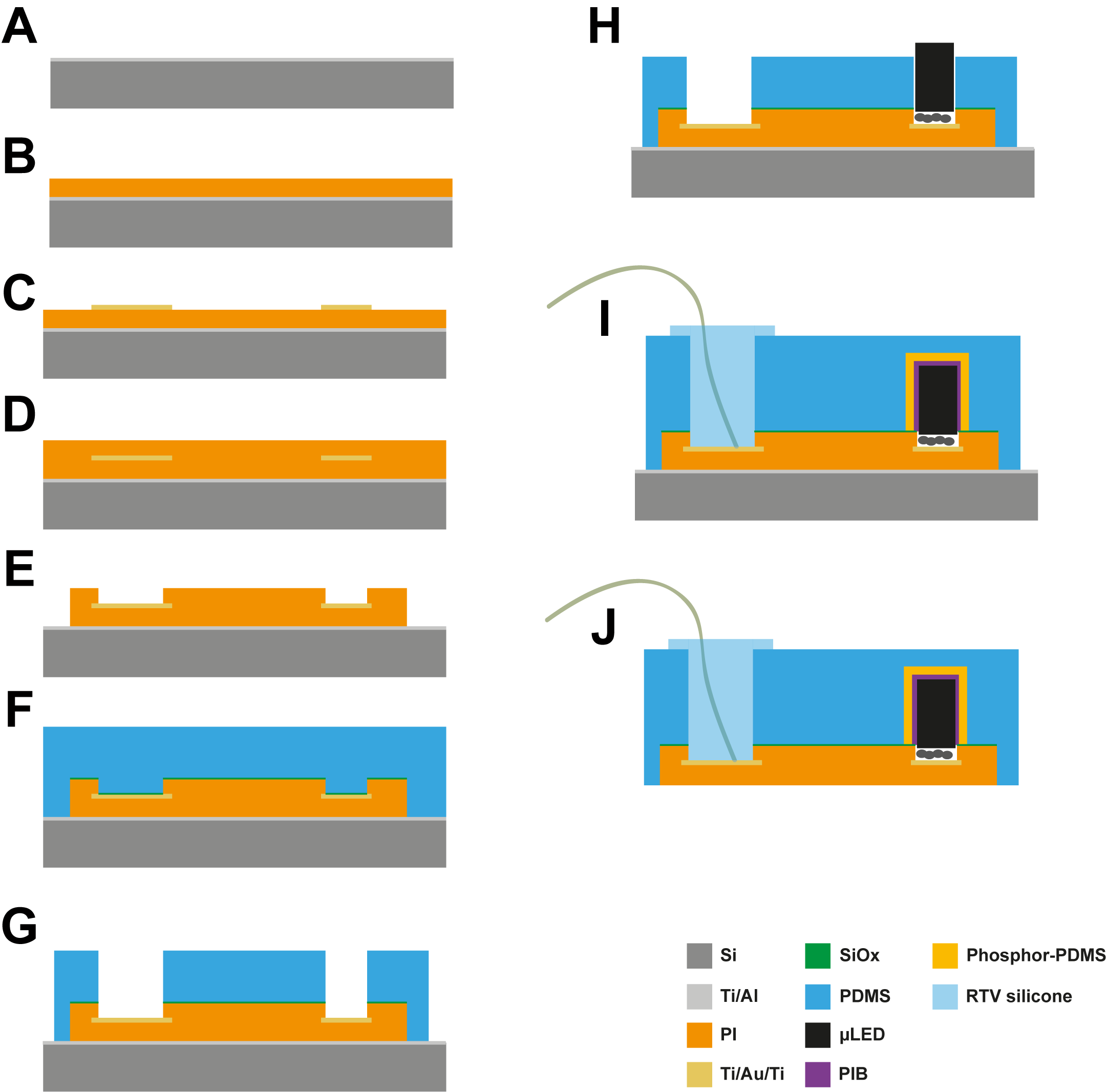

Supplement: Supplementary file 1 — Supplemental Figure S1. Simplified microfabrication process flow. (A) A 4″ Si wafer with a Ti/Al (10/100 nm) sacrificial layer is used as a carrier. (B) Spin‐coating and baking of a bottom 3‐μm layer of PI. (C) Sputtering and etching of Ti/Au/Ti interconnects (wet etching and RIE). (D) Spin‐coating and baking of a top 3‐μm layer of PI. (E) Patterning of PI outline and opening contact sites through O2 RIE. (F) Sputtering of 25 nm SiO x adhesion layer and coating of 35 μm PDMS. (G) SiOx and PDMS RIE etching to pattern outline and open contact sites. (H) Solder paste stamping and μLED placement. (I) Encapsulation of μLED with PIB, yellow phosphor‐PDMS and PDMS, copper wire soldering, and RTV encapsulation. (J) Anodic Al dissolution for structure release from the wafer. [file BTM2-10-e70034-s001.tif]

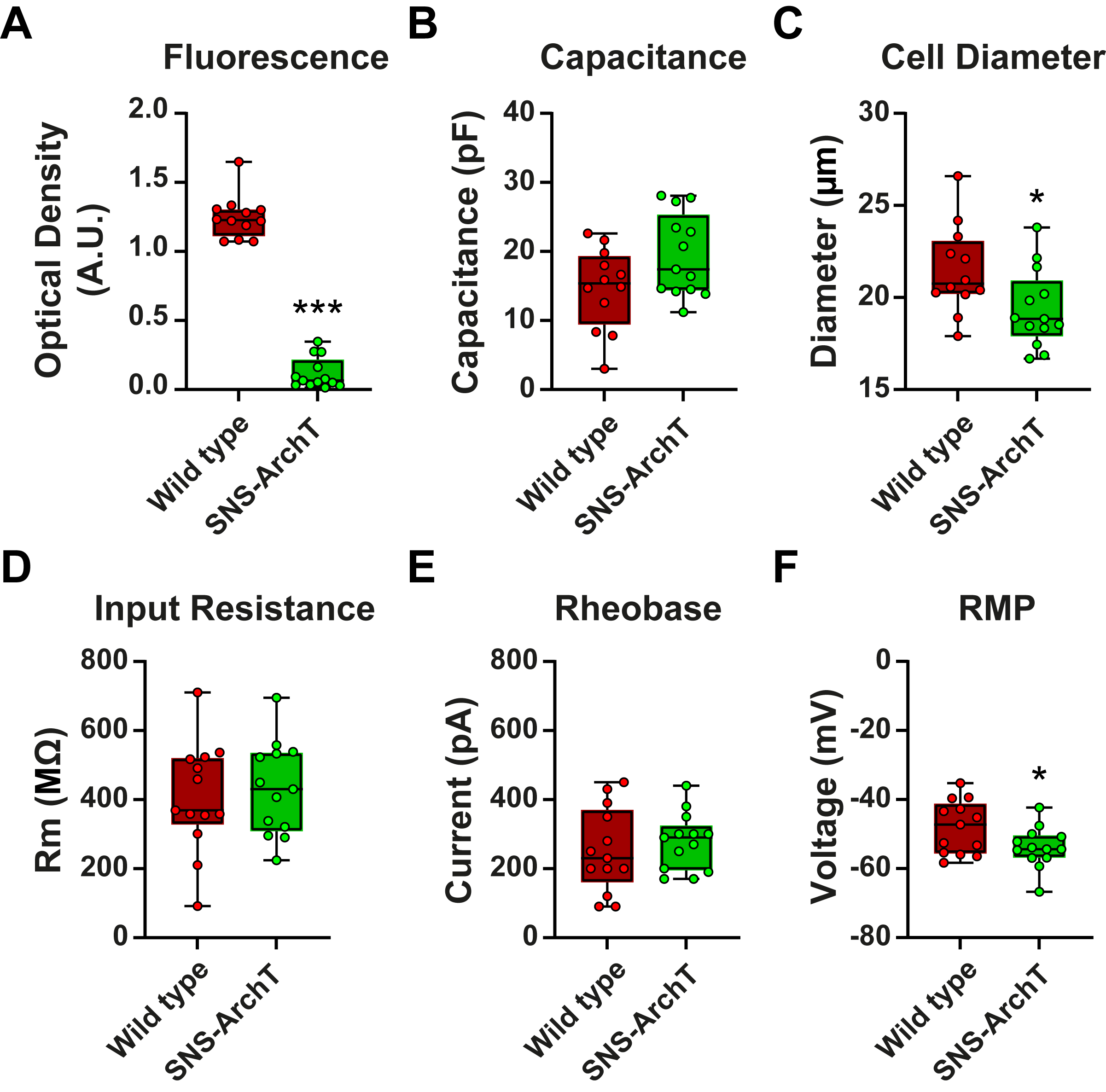

Supplement: Supplementary file 2 — Supplemental Figure S2. Patch clamp recording of dissociated DRG neurons expressing ArchT. (A) Quantification of the fluorescence of the recorded neurons from SNS‐ArchT (green) or control (red) littermate mice (N = 12–13 cells/group. Unpaired t‐test with welch correction, t(19.76) = 20.62, *** p < 0.0001). The lower the optical density is the more fluorescent the cells are. (B) Quantified capacitance (in pF) of the recorded dissociated DRG neurons from SNS‐ArchT (green) or control (red) littermate mice (N = 12–13 cells/group. Unpaired t‐test with welch correction, t(22.86) = 2.009, p = 0.0565). The lower the capacitance is the smaller the recorded cells are considered. (C) Quantified cell diameter of the recorded dissociated DRG neurons from SNS‐ArchT (green) or control (red) littermate mice (N = 12–13 cells/group. Unpaired t‐test with welch correction, t(22.09) = 2.335, * p = 0.029). The difference is probably due to the selection of cells: fluorescent ones belong to a majority of small DRG neurons, while in control littermates the selection is random (no fluorescence). (D) Quantified input resistance of the recorded dissociated DRG neurons from SNS‐ArchT (green) or control (red) littermate mice (N = 12–13 cells/group. Unpaired t‐test with Welch correction, t(22.39) = 0.4298, p = 0.6713). (E) Quantified rheobase of the recorded dissociated DRG neurons from SNS‐ArchT (green) or control (red) littermate mice (N = 12–13 cells/group. Unpaired t‐test with welch correction, t(21.05) = 0.6215, p = 0.541). (F) Quantified resting membrane potential (RMP, in mV) of the recorded dissociated DRG neurons from SNS‐ArchT (green) or control (red) littermate mice (N = 12–13 cells/group. Unpaired t‐test with welch correction, t(22.48) = 2.159, * p = 0.0418). [file BTM2-10-e70034-s006.tif]

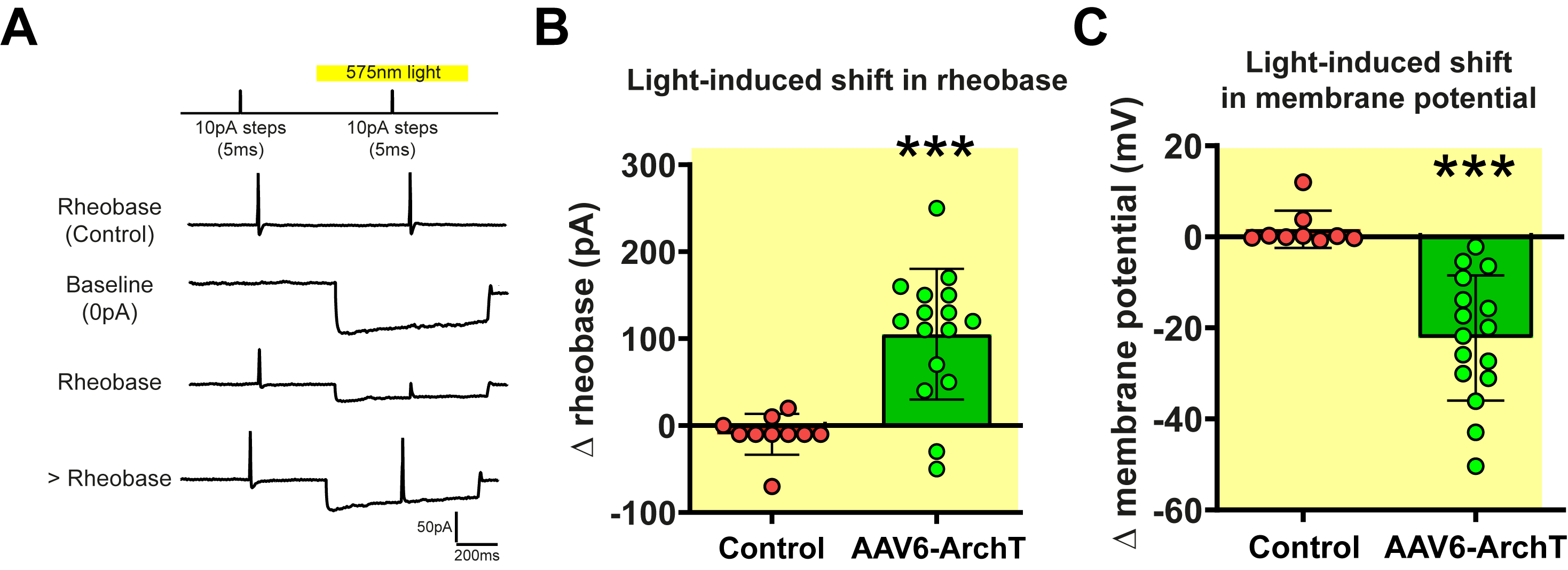

Supplement: Supplementary file 3 — Supplemental Figure S3. Optogenetic inhibition of dissociated DRG neurons in vitro transduced with an AAV6‐CMV‐ArchT‐GFP vector. (A) Representative traces of recordings in the different conditions. Current clamp protocol consists of two depolarising 5 ms steps increased by 10 pA after every sweep to determine the rheobase, without and then with yellow inhibitory light (575 nm). (B), (C) Quantified effects of optogenetic inhibition on (B) the rheobase (N = 10–16 cells/group; unpaired t‐test with welch correction, t(19.29) = 5.687, *** p < 0.001) and (C) the membrane potential (N = 9–16 cells/group; unpaired t‐test with welch correction, t(19.2) = 6.454, *** p < 0.001) in GFP+ DRG neurons (green), using the GFP− DRG neurons as control (red). [file BTM2-10-e70034-s003.tif]

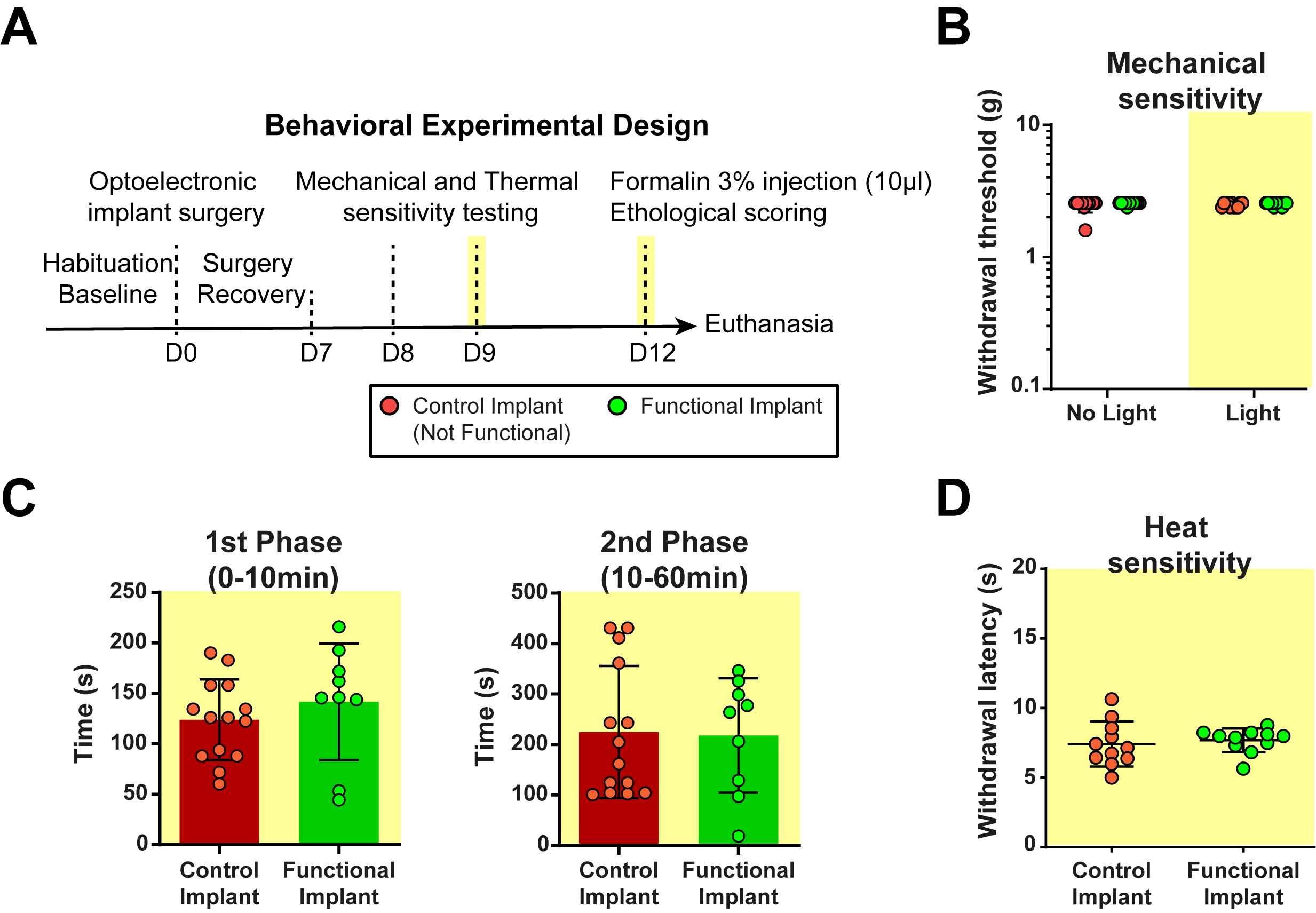

Supplement: Supplementary file 4 — Supplemental Figure S4. Optogenetic stimulation protocol and experimental design. (A) Experimental Design. Following the implantation, the animals went through a week of recovery and were tested for mechanical and thermal sensitivity without light first, then with light on (yellow lines). Then, they received an intraplantar injection of formalin 3%, and spontaneous paw withdrawal was measured for 80 min with yellow light stimulation (same stimulation parameters as previously: 20 s light train pulses, 1 Hz, 80% duty‐cycle, 1 s inter‐train interval). The control group were either transgenic mice implanted with non‐functional implants, or control littermates implanted with a functional implant. Both groups behaved similarly and were therefore pooled. (B) Mechanical sensitivity measured, using the standard Von Frey up and down method, on the right non‐implanted paw from functional (green) or control conditions (red) (N = 11/group. ANOVA two‐way repeated measures, no significant effect of the Stimulation factor p = 0.794, the Group factor p = 0.3188 nor Interactions p = 0.8437). (C) Measurement of the total paw withdrawal duration (in s), during the acute phase (0–10 min), or the latest phase (10–60 min), after intraplantar formalin injection (N = 9‐10/group. Unpaired t‐test with welch correction, no significant differences: (0–10 min) p = 0.3562; (10–60 min) p > 0.99). (D) Thermal sensitivity toward noxious stimulation, tested on the Hargreaves test, on the right non‐implanted paw (N = 11/group. Unpaired t‐test with welch correction, non‐significant differences p = 0.6358). [file BTM2-10-e70034-s004.tif]
