# Supplementary material for: Optogenetic modulation of peripheral nociceptive neurons with biocompatible optoelectronic implants
Source: Bioeng Transl Med. 2025 Jun 26;10(4):e70034. doi: 10.1002/btm2.70034 (PMC12284439; doi:10.1002/btm2.70034)
Supplement: Supplementary file 5 — Supplemental Data Table 1.RRID and source references of the resources used in the current study. [file BTM2-10-e70034-s002.docx]

| **Supplemental Data Table 1: Products references RRID** | | | |
| --- | --- | --- | --- |
| Reagent or resource | Source (Company, City, State, Country) | Catalogue Number | RRID (Resource Identification Portal)/MGI (Mouse Genome Informatics) |
| **Mice** |  |  |  |
| Mouse line STOP-floxed ArchT-eGFP | Jackson Laboratory, 600 Main Street , Bar Harbor, ME 04609 | B6.Cg-Gt(ROSA)26Sortm40.1(CAG-aop3/EGFP)Hze/J | RRID:IMSR_JAX:021188 |
| Mouse line SNS-Cre | Rohini Kuner | Tg(Scn10a-cre)1Rkun | MGI Cat# 3042874, RRID:MGI:3042874 |
| **Antibodies** |  |  |  |
| Donkey Cy3 anti-mouse | Jackson ImmunoResearch, Ely, UK | 715-165-151 | RRID:AB_2315777 |
| Donkey Cy3 anti-rabbit | Jackson ImmunoResearch, Ely, UK | 711-165-152 | RRID:AB_2307443 |
| Isolectin 4 | Vector Laboratories, Newark, CA, USA | B-1205 | RRID:AB_2314664 |
| Mouse anti-NF200 | Sigma-Aldrich Inc., Saint-Louis, Missouri (MO), USA | 0142 | RRID:AB_477257 |
| Rabbit anti-CGRP | Peninsula Laboratories LLC, San Carlos, CA, USA | T-4032 | RRID:AB_518147 |
| Rabbit anti-Peripherin | Chemicon International Inc. | AB1530 | RRID:AB_90725 |
| Streptavidine AMCA | Jackson ImmunoResearch, Ely, UK | 016-150-084 | RRID:AB_2337243 |
| **Material/Equipment for Implant fabrication** |  |  |  |
| Au wet etching | MicroChemicals GmbH, Ulm, Germany | TechniEtch™ACI2 |  |
| Copper wires | Cooner wires, Chatsworth, CA, USA | CZ1103 |  |
| Cyclohexane | Sigma-Aldrich Inc., Saint-Louis, Missouri (MO), USA | cyclohexane, 110-82-7 |  |
| Dosage instrument | Abatech, La Chaux-de-Fonds, Switzerland | KDG1000 |  |
| Keithely source measure unit instrument | Tektronix UK Ltd, Bershire, UK | Keithely 2400 sourcemeter |  |
| Magnetron sputtering instrument | Alliance Concept, Annecy, France | AC450 |  |
| Microscope slide power sensor | Thorlabs, Germany (European Hub) | photodiode S170C |  |
| PDMS | Dow Corning, USA | Sylgard 184 |  |
| Photolitography (2um) | MicroChemicals GmbH, Ulm, Germany | AZ 1512 HS |  |
| Photolitography (40um) | MicroChemicals GmbH, Ulm, Germany | AZ 40XT |  |
| Photolitography (8um) | MicroChemicals GmbH, Ulm, Germany | AZ 10XT |  |
| Pick-and-place tool | Microtechnic, Marcoussis, France | JFP PP-One |  |
| Polyimide | HD Microsystems GmbH | PI2611 |  |
| polyisobutylen | BASF, Ludwigshafen, Germany | PIB, Oppanol |  |
| Power and energy meter console | Thorlabs, Germany (European Hub) | power meter PM100D |  |
| Silicone | Dow Corning, USA | DOWSIL 734 |  |
| Sn/Bi/Ag solder paste (SMDLTLFP10T5, Chipquik | Chipquik Inc., DigiKey, MN, USA | SMDLTLFP10T5 |  |
| Ti Reactive Ion Etching | Corial, Bernin, France | RIE: 201RL |  |
| Unsheathed fine gage thermocouple | Omega Engineering Inc., Norwalk CT, USA | CHAL-002 |  |
| Yellow phosphor | PhosphorTech Corporation, Kennesaw, Georgia, USA | Y555 |  |
| μ-LED bare dies | Cree Inc., Durham, NC, USA | 240 x 320 x 140 μm3, 470 nm, DA2432 |  |
| **Material/Equipment** |  |  |  |
| Borosilicate Pipettes | Sutter instrument, Novato, California (CA), USA | #BF150-86-7.5 |  |
| CoolLED pE-340 fura | CoolLED Ltd, Andover, UK | #pE-340 fura |  |
| Coverslips 12 mm | VWR, Radnor, Pennsylvania (PA), USA | #631-1577 |  |
| Dental acrylic | Kulzer GmbH, Hanau, Germany | Paladur |  |
| Digidata 1440A | Molecular Devices, San Jose, California (CA), USA | #1440A |  |
| Falcon® 25cm² Rectangular Canted Neck Cell Culture Flask with Vented Cap | Corning Inc., Corning, New-York (NY), USA | #353108 |  |
| Falcon® 6-well Clear Flat Bottom TC-treated Multiwell Cell Culture Plate | Corning Inc., Corning, New-York (NY), USA | #353224 |  |
| Falcon® P100 cell culture dishes | Corning Inc., Corning, New-York (NY), USA | #353003 |  |
| Gilson minipuls suction system | Gilson, Middleton, WI, USA |  |  |
| Glue Instant Adhesive | Henkel AG & Co. KGaA, Düsseldorf, Germany | Loctite 401 |  |
| Gluture topical | World Precision Instruments, Sarasota, FL, USA | 503763 |  |
| Gold Plated screws (diameter 1.1mm) |  |  |  |
| H4 platinum/iridium wire | World Precision Instruments, Sarasota, FL, USA | MF-200 H4 |  |
| LED eGFP pE-300 filterset | CoolLED Ltd, Andover, UK | #E3990113, Exciter: 460/30; Emitter: 520/40 |  |
| Confocal microscope | Zeiss, Oberkochen, Germany | LSM710 Quasar |  |
| Male-female EEG connector | Conrad Electronic AG, Wollerau, Switzerland | 2103944 - 62 |  |
| Male-male EEG connector | Conrad Electronic AG, Wollerau, Switzerland | 741090 - 62 |  |
| MF200-2 microforge | World Precision Instruments, Sarasota, FL, USA | MF200-2 |  |
| Multiclamp Axon Amplifier 700B | Molecular Devices, San Jose, California (CA), USA | #700B |  |
| Nalgene 4 mm syringe filters | Thermo Fisher Scientific Inc., Waltham, Massachusetts (MA), USA | 176-0020 |  |
| Olympus BX51WI fluorescent microscope | Olympus, Tokyo, Japan | BX51WI |  |
| ORCAFlash2.8, model C11440-10C | Hamamatsu Photonics, Shizuoka, Japan | #820504 |  |
| Osmometer 3320 | Advanced Instruments Inc | 3320 |  |
| Perfusion valve controller VC-6 | Warner Instrument, Hamden, CT, USA | VC-6 |  |
| Plantar Radiant heat | IITC Life Science Inc., CA, USA | #390G |  |
| SevenCompact S210 | Mettler Toledo, Columbus, OH, USA | S210 |  |
| Suture filaments threads Vicryl 5-0 | Ethicon | K880H |  |
| Suture filaments threads Vicryl 6-0 | Ethicon | K802H |  |
| TH4 halogen lamp power supply unit | Olympus, Tokyo, Japan | TH4 |  |
| Von Frey | Bioseb, Vitrolles, France | BIO-VF-M |  |
| W30S-LED Revelation III | LW Scientific, Lawrenceville, GA, USA | W30S |  |
| Wires Toron PVC | Distrelec | 511343 |  |
| Wound Clips reflex 7mm | Stoelting | 59035 |  |
| Zeiss Axio Scan.Z1 slide scanner | Zeiss, Oberkochen, Germany | AxioPlan |  |
| **Software** |  |  |  |
| Fiji (ImageJ Software) | Schindelin et al., 2012 |  | Fiji, RRID:SCR_002285 |
| GraphPad Prism 9 Software | GraphPad Software Inc., San Diego, California (CA), USA |  | GraphPad Prism, RRID:SCR_002798 |
| Olympus CellSens v3.2 acquisition software | Olympus, Tokyo, Japan | CellSens v3.2 | RRID:SCR_014551 |
| pClamp 10 Software | Molecular Devices, San Jose, California (CA), USA |  | pClamp, RRID:SCR_011323 |
| R studio | R software Open source | RStudio 2023.06.0+421 | RRID:SCR_000432 |
| **Reagents** |  |  |  |
| B27 Plus supplement | Thermo Fisher Scientific Inc., Waltham, Massachusetts (MA), USA | A3582801 |  |
| Betadine | MundiPharma |  |  |
| CaCl2 (Calcium chloride dihydrate) | Merck Millipore, Burlington, Massachusetts (MA), USA | #102382 |  |
| Carprofen | Norbrook® Laboratories Limited, Newry, Northern Ireland | Rimadyl |  |
| Collagenase A | Roche, Basel, Switzerland | 10103578001 |  |
| Dispase II | Roche, Basel, Switzerland | 4942078001 |  |
| DMEM | Gibco, Billings, MT, USA | 41965-039 |  |
| EGTA (Ethylene Glycol Tetra Acetic Acid) | Sigma-Aldrich Inc., Saint-Louis, Missouri (MO), USA | #E4378 |  |
| formalin | Thermo Fisher Scientific Inc., Waltham, Massachusetts (MA), USA | 119690010 |  |
| Hanks' Balanced Salt solution | Sigma-Aldrich Inc., Saint-Louis, Missouri (MO), USA | H6648-500ML |  |
| HEPES | AppliChem, Darmstadt, Germany | #A1069 |  |
| Isoflurane | Piramal, Mumbai, Maharashtra, India | G45C19A |  |
| KCl (Potassium Chloride) | Sigma-Aldrich Inc., Saint-Louis, Missouri (MO), USA | #P9333 |  |
| MgCl2-6H2O (Magnesium chloride hexahydrate) | Merck Millipore, Burlington, Massachusetts (MA), USA | #105832 |  |
| Mowiol 4–88 medium | Merck, Kenilworth, NJ, USA | #475904 |  |
| Na2-ATP (Adenosine 5′-triphosphate disodium salt hydrate) | Sigma-Aldrich Inc., Saint-Louis, Missouri (MO), USA | #A2383 |  |
| NaCl (Sodium Chloride) | Sigma-Aldrich Inc., Saint-Louis, Missouri (MO), USA | #S9625 |  |
| NaOH (Sodium hydroxide) | Sigma-Aldrich Inc., Saint-Louis, Missouri (MO), USA | #S8045 |  |
| Neurobasal Plus Medium | Thermo Fisher Scientific Inc., Waltham, Massachusetts (MA), USA | A3582901 |  |
| Normal goat serum | Vector Laboratories, Newark, CA, USA | S-1000 |  |
| Ophtalmic gel | Vita-Pos, Pharma medica |  |  |
| Paracetamol | UPSA SAS, Rueil-Malmaison, France | Dafalgan |  |
| Paraformaldehyde | Sigma-Aldrich Inc., Saint-Louis, Missouri (MO), USA | P6148 |  |
| Penicillin/Streptomycin (P/S) | Sigma-Aldrich Inc., Saint-Louis, Missouri (MO), USA | #P0781 |  |
| Pentobarbital | Streuli Pharma, USA | V102013 |  |
| Phosphate buffered saline (PBS) pH7.4 | Thermo Fisher Scientific Inc., Waltham, Massachusetts (MA), USA | #10010 |  |
| Poly-D-Lysine | Sigma-Aldrich Inc., Saint-Louis, Missouri (MO), USA | #P7886 |  |
| Strepatvidin Sepharose High Performance Beads | GE Healthcare, Chicago, Illinois (IL), USA? | #17-5113-01 |  |
| Sucrose (D+ Saccharose) | AppliChem, Darmstadt, Germany | #A2211 |  |
| Tris | AppliChem, Darmstadt, Germany | #A1086 |  |
| Triton X-100 | Sigma-Aldrich Inc., Saint-Louis, Missouri (MO), USA | #T9284 |  |
