# Supplementary material for: Optogenetic modulation of peripheral nociceptive neurons with biocompatible optoelectronic implants
Source: Bioeng Transl Med. 2025 Jun 26;10(4):e70034. doi: 10.1002/btm2.70034 (PMC12284439; doi:10.1002/btm2.70034)
Supplement: Supplementary file 6 — Supplemental Data Table 2. Statistical table. Each row represents a dataset and the corresponding statistical tests, statistical values, p‐values, and effect size factors (generalized eta square η 2 or Cohen's d). [file BTM2-10-e70034-s005.docx]

| **Extended Data Table: Statistical Analysis** | | | | | | | |  |  |  |  |
| --- | --- | --- | --- | --- | --- | --- | --- | --- | --- | --- | --- |
| **Figure** | **Data Type** | **Statistical test Type** | **N** | **Stat-value** | **P-value (or p.adj.)** | **p.adj.signif** | **Effect size (Generalized eta squared η^2^_G_ / Cohen d)** |  |  |  |  |
|  |  |  |  |  |  |  |  |  |  |  |  |
| Figure 1E | Mechanical sensitivity evoked with VF filaments after implant, SNI or sham surgery (left paw operated) | ANOVA Two-way RM (Factors=Surgery~TimePostInjury) | Implant=14 SNI=12 Sham=12 | Time Post Injury: F(5, 175)=13.8 Surgery: F(2, 35)=8.47 Interaction: F(10, 175)=2.29 | Time Post Injury: p<0.001 Surgery: p<0.01 Interaction: p=0.015 |  | Time Post Injury: η^2^_G_= 0.230 Surgery: η^2^_G_= 0.105 Interaction: η^2^_G_= 0.090 |  |  |  |  |
|  |  | Tukey Post hoc analysis | BL | Implant vs. Sham | p=0.813 | ns |  | Mean and SD | BL | Implant | 1.902 ± 0.731 |
|  |  |  |  | Implant vs. SNI | p=0.978 | ns |  |  |  | Sham | 1.718 ±0.746 |
|  |  |  |  | Sham vs. SNI | p=0.916 | ns |  |  |  | SNI | 1.842 ±0.871 |
|  |  |  | D2 | Implant vs. Sham | p=0.349 | ns |  |  | D2 | Implant | 1.065 ±0.818 |
|  |  |  |  | Implant vs. SNI | p=0.211 | ns |  |  |  | Sham | 0.649 ±0.731 |
|  |  |  |  | Sham vs. SNI | p=0.955 | ns |  |  |  | SNI | 0.559 ±0.665 |
|  |  |  | D4 | Implant vs. Sham | p=0.991 | ns |  |  | D4 | Implant | 0.705 ±0.819 |
|  |  |  |  | Implant vs. SNI | p=0.605 | ns |  |  |  | Sham | 0.665 ±0.477 |
|  |  |  |  | Sham vs. SNI | p=0.706 | ns |  |  |  | SNI | 0.418 ±0.528 |
|  |  |  | D7 | Implant vs. Sham | p=0.057 | ns |  |  | D7 | Implant | 0.752 ±0.649 |
|  |  |  |  | Implant vs. SNI | p=0.281 | ns |  |  |  | Sham | 1.441 ±0.84 |
|  |  |  |  | Sham vs. SNI | p=0.0008 | *** |  |  |  | SNI | 0.295 ±0.382 |
|  |  |  | D14 | Implant vs. Sham | p=0.29 | ns |  |  | D14 | Implant | 0.922 ±0.989 |
|  |  |  |  | Implant vs. SNI | p=0.027 | * |  |  |  | Sham | 1.373 ±0.967 |
|  |  |  |  | Sham vs. SNI | p=0.0003 | *** |  |  |  | SNI | 0.145 ±0.187 |
|  |  |  | D21 | Implant vs. Sham | p=0.939 | ns |  |  | D21 | Implant | 1.127 ±1.035 |
|  |  |  |  | Implant vs. SNI | p=0.017 | * |  |  |  | Sham | 1.229 ±0.876 |
|  |  |  |  | Sham vs. SNI | p=0.0088 | ** |  |  |  | SNI | 0.301 ±0.72 |
|  |  |  | Implant | BL vs. D2 | p=0.042 | * |  |  |  |  |  |
|  |  |  |  | BL vs. D4 | p=0.024 | * |  |  |  |  |  |
|  |  |  |  | BL vs. D7 | p=0.0018 | ** |  |  |  |  |  |
|  |  |  |  | BL vs. D14 | p=0.096 | ns |  |  |  |  |  |
|  |  |  |  | BL vs. D14 | p=0.332 | ns |  |  |  |  |  |
|  |  |  | SNI | BL vs. D2 | p=0.006 | ** |  |  |  |  |  |
|  |  |  |  | BL vs. D4 | p=0.005 | ** |  |  |  |  |  |
|  |  |  |  | BL vs. D7 | p=0.002 | ** |  |  |  |  |  |
|  |  |  |  | BL vs. D14 | p=0.0006 | *** |  |  |  |  |  |
|  |  |  |  | BL vs. D14 | p<0.0001 | *** |  |  |  |  |  |
|  |  |  | Sham | BL vs. D2 | p=0.038 | * |  |  |  |  |  |
|  |  |  |  | BL vs. D4 | p=0.005 | ** |  |  |  |  |  |
|  |  |  |  | BL vs. D7 | p=0.923 | ns |  |  |  |  |  |
|  |  |  |  | BL vs. D14 | p=0.848 | ns |  |  |  |  |  |
|  |  |  |  | BL vs. D14 | p=0.518 | ns |  |  |  |  |  |
|  |  |  |  |  |  |  |  |  |  |  |  |
| Figure 3B | Shift in Rheobase induced by yellow light recorded by current clamp recordings | Unpaired t-test with Welch correction | Control littermates=13 SNS-ArchT=13 | t_(12.59)_ =4.46 | p=0.0007 | *** | Cohen d = -1.694 | Mean and SD |  | Control littermates | -20 ±11.55 |
|  |  |  |  |  |  |  |  |  |  | SNS-ArchT | 72.31 ±73.73 |
| Figure 3C | Shift in membrane potential induced by yellow light recorded by current clamp recordings | Unpaired t-test with Welch correction | Control littermates=13 SNS-ArchT=13 | t_(12.38)_ =7.696 | p<0.0001 | *** | Cohen d = 2.9232 | Mean and SD |  | Control littermates | 1.48 ±0.949 |
|  |  |  |  |  |  |  |  |  |  | SNS-ArchT | -14.83 ±7.58 |
|  |  |  |  |  |  |  |  |  |  |  |  |
| Supplementary Figure S2A | Optical Density of recorded cells | Unpaired t-test with Welch correction | Control littermates=12 SNS-ArchT=13 | t_(19.76)_ =20.62 | p<0.0001 | *** | Cohen d = 8.0923 | Mean and SD |  | Control littermates | 1.247 ±0.1568 |
|  |  |  |  |  |  |  |  |  |  | SNS-ArchT | 0.1159 ±0.1118 |
| Supplementary Figure S2B | Capacitance of recorded cells | Unpaired t-test with Welch correction | Control littermates=12 SNS-ArchT=13 | t_(22.86)_ =2.009 | p=0.0565 | ns | Cohen d = -0.7776 | Mean and SD |  | Control littermates | 14.66 ±5.894 |
|  |  |  |  |  |  |  |  |  |  | SNS-ArchT | 19.42 ±5.93 |
| Supplementary Figure S2C | Diameter of recorded cells | Unpaired t-test with Welch correction | Control littermates=12 SNS-ArchT=13 | t_(22.09)_ =2.335 | p=0.029 | * | Cohen d = 0.90835 | Mean and SD |  | Control littermates | 21.48 ±2.391 |
|  |  |  |  |  |  |  |  |  |  | SNS-ArchT | 19.36 ±2.119 |
| Supplementary Figure S2D | Input resistance of recorded cells | Unpaired t-test with Welch correction | Control littermates=13 SNS-ArchT=13 | t_(23.39)_ =0.4298 | p=0.6713 | ns | Cohen d = -0.16327 | Mean and SD |  | Control littermates | 406.1 ±158.8 |
|  |  |  |  |  |  |  |  |  |  | SNS-ArchT | 431 ±134.9 |
| Supplementary Figure S2E | Rheobase of recorded cells | Unpaired t-test with Welch correction | Control littermates=13 SNS-ArchT=13 | t_(21.05)_ =0.6215 | p=0.541 | ns | Cohen d = -0.23607 | Mean and SD |  | Control littermates | 252.3 ±122.1 |
|  |  |  |  |  |  |  |  |  |  | SNS-ArchT | 277.7 ±82.38 |
| Supplementary Figure S2F | Resting Membrane Potential of recorded cells | Unpaired t-test with Welch correction | Control littermates=13 SNS-ArchT=13 | t_(22.48)_ =2.159 | p=0.0418 | * | Cohen d = 0.81997 | Mean and SD |  | Control littermates | -48.04 ±7.673 |
|  |  |  |  |  |  |  |  |  |  | SNS-ArchT | -53.82 ±5.881 |
|  |  |  |  |  |  |  |  |  |  |  |  |
| Supplementary Figure S3B | Shift in Rheobase induced by yellow light recorded by current clamp recordings in DRG neurons transduced with an AAV6-CMV-ArchT-GFP | Unpaired t-test with Welch correction | Control cells=9 AAV6-CMV-ArchT=16 | t_(19.29)_ =5.687 | p<0.0001 | *** | Cohen d = 1.821 | Mean and SD |  | Control littermates | -10 ±23.57 |
|  |  |  |  |  |  |  |  |  |  | AAV6-CMV-ArchT-GFP | 105 ±75.19 |
| Supplementary Figure S3C | Shift in membrane potential induced by yellow light recorded by current clamp recordings in DRG neurons transduced with an AAV6-CMV-ArchT-GFP | Unpaired t-test with Welch correction | Control cells=9 AAV6-CMV-ArchT=16 | t_(19.2)_ =6.454 | p<0.0001 | *** | Cohen d = -2.031 | Mean and SD |  | Control littermates | 1.67 ±4.1 |
|  |  |  |  |  |  |  |  |  |  | AAV6-CMV-ArchT-GFP | -22.21 ±13.76 |
|  |  |  |  |  |  |  |  |  |  |  |  |
| Figure 4B | Mechanical sensitivity evoked with 4g-VF filaments (Implanted Left paw) | ANOVA Two-way RM (Factors=Stimulation~Genotype) | Functionnal=11 Control=11 | Stimulation: F(1, 20)=26.94 Group: F(1, 20)=5.053 Interaction: F(1, 20)=14.63 | Stimulation: p<0.0001 Group: p=0.036 Interaction: p=0.0011 |  | Stimulation: η^2^_G_= 0.301 Group: η^2^_G_= 0.147 Interaction: η^2^_G_= 0.190 | Mean and SD | No Stimulation | Control | 78.18 ± 12.5 |
|  |  | Sidak's multiple comparison Post hoc analysis test | No Stimulation | Functionnal vs. Control | p=0.9419 | ns |  |  |  | Functionnal | 80.0 ±10.0 |
|  |  |  | Stimulation | Functionnal vs. Control | p=0.0005 | *** |  |  | Stimulation | Control | 73.64 ± 17.48 |
|  |  |  |  |  |  |  |  |  |  | Functionnal | 50.0 ±14.14 |
|  |  |  |  |  |  |  |  |  |  |  |  |
|  |  |  | Control | No Stimulation vs. Stimulation | p=0.5718 | ns |  |  |  |  |  |
|  |  |  | Functionnal | No Stimulation vs. Stimulation | p<0.0001 | *** |  |  |  |  |  |
| Figure 4C | Thermal sensitivity evoked with radiant heat beam (Implanted Left paw) | Unpaired t-test with Welch correction | Functionnal=11 Control=11 | t_(17.68)_ =2.513 | p=0.0219 | * | Cohen d = -1.03 | Mean and SD | Control | 7.022 ± 2.383 |  |
|  |  |  |  |  |  |  |  |  | Functionnal | 9.209 ±1.63 |  |
|  |  |  |  |  |  |  |  |  |  |  |  |
| Figure 4D | Mechanical sensitivity evoked with VF filaments up and down method (Implanted Left paw) | ANOVA Two-way RM (Factors=Stimulation~Genotype) | Functionnal=11 Control=11 | Stimulation: F(1, 20)=0.345 Group: F(1, 20)=0.3277 Interaction: F(1, 20)=0.0030 | Stimulation: p=0.5872 Group: p=0.5734 Interaction: p=0.9570 |  | Stimulation: η^2^_G_= 0.007 Group: η^2^_G_= 0.009 Interaction: η^2^_G_= 0.00066 | Mean and SD | Baseline | Control | 2.152 ±0.6312 |
|  |  |  |  |  |  |  |  |  |  | Functionnal | 2.478 ±0.095 |
|  |  |  |  |  |  |  |  |  | No Stimulation | Control | 2.266 ± 0.5735 |
|  |  |  |  |  |  |  |  |  |  | Functionnal | 2.162 ±0.5885 |
|  |  |  |  |  |  |  |  |  | Stimulation | Control | 2.341 ±0.4493 |
|  |  |  |  |  |  |  |  |  |  | Functionnal | 2.253 ±0.4947 |
| Figure 4E | Paw withdrawal duration after Formalin intraplantar injection | ANOVA Two-way RM (Factors=Time Post Injection~Genotype) | Functionnal=9 Control=10 | Time Post Injection: F(5.16, 87.72)=27.37 Group: F(1, 17)=0.3298 Interaction: F(16, 272)=1.237 | Time Post Injection: p<0.0001 Group: p=0.5733 Interaction: p=0.2393 |  | Time Post Injection: η^2^_G_= 0.553 Group: η^2^_G_= 0.004 Interaction: η^2^_G_= 0.055 | Mean and SD | Control | 0-5min | 111.84 ±39.14 |
|  |  |  |  |  |  |  |  |  |  | 5-10min | 10.85 ±9.41 |
|  |  |  |  |  |  |  |  |  |  | 10-15min | 11.21 ±12.18 |
|  |  |  |  |  |  |  |  |  |  | 15-20min | 57.98 ±46.66 |
|  |  |  |  |  |  |  |  |  |  | 20-25min | 48.65 ±29.51 |
|  |  |  |  |  |  |  |  |  |  | 25-30min | 25.87 ±19.53 |
|  |  |  |  |  |  |  |  |  |  | 30-35min | 35.07 ±58.13 |
|  |  |  |  |  |  |  |  |  |  | 35-40min | 13.1 ±17.82 |
|  |  |  |  |  |  |  |  |  |  | 40-45min | 11.99 ±21.69 |
|  |  |  |  |  |  |  |  |  |  | 45-50min | 6.73 ±10.05 |
|  |  |  |  |  |  |  |  |  |  | 50-55min | 3.12 ±3.52 |
|  |  |  |  |  |  |  |  |  |  | 55-60min | 10.66 ±28.03 |
|  |  |  |  |  |  |  |  |  |  | 60-65min | 2.65 ±6.72 |
|  |  |  |  |  |  |  |  |  |  | 65-70min | 1.68 ±2.00 |
|  |  |  |  |  |  |  |  |  |  | 70-75min | 5.28 ±12.7 |
|  |  |  |  |  |  |  |  |  |  | 75-80min | 8.73 ±17.1 |
|  |  |  |  |  |  |  |  |  | Functionnal | 0-5min | 125.46 ±48.24 |
|  |  |  |  |  |  |  |  |  |  | 5-10min | 16.18 ±14.63 |
|  |  |  |  |  |  |  |  |  |  | 10-15min | 18.36 ±23.52 |
|  |  |  |  |  |  |  |  |  |  | 15-20min | 26.86 ±31.53 |
|  |  |  |  |  |  |  |  |  |  | 20-25min | 33.53 ±20.48 |
|  |  |  |  |  |  |  |  |  |  | 25-30min | 27.85 ±19.07 |
|  |  |  |  |  |  |  |  |  |  | 30-35min | 32.37 ±27.54 |
|  |  |  |  |  |  |  |  |  |  | 35-40min | 27.04 ±26.53 |
|  |  |  |  |  |  |  |  |  |  | 40-45min | 15.37 ±18.26 |
|  |  |  |  |  |  |  |  |  |  | 45-50min | 13.63 ±11.85 |
|  |  |  |  |  |  |  |  |  |  | 50-55min | 10.54 ±11.7 |
|  |  |  |  |  |  |  |  |  |  | 55-60min | 12.39 ±20.13 |
|  |  |  |  |  |  |  |  |  |  | 60-65min | 23.64 ±40.39 |
|  |  |  |  |  |  |  |  |  |  | 65-70min | 10.8 ±11.02 |
|  |  |  |  |  |  |  |  |  |  | 70-75min | 9.64 ±20.97 |
|  |  |  |  |  |  |  |  |  |  | 75-80min | 10.49 ±21.06 |
|  |  |  |  |  |  |  |  |  |  |  |  |
| Supplementary Figure S4B | Mechanical sensitivity evoked with VF filaments up and down method (Non-Implanted Right paw) | ANOVA Two-way RM (Factors=Stimulation~Genotype) | Functionnal=11 Control=11 | Stimulation: F(1, 20)=0.0029 Group: F(1, 20)=2.342 Interaction: F(1, 20)=0.1410 | Stimulation: p=0.9573 Group: p=0.1416 Interaction: p=0.7113 |  | Stimulation: η^2^_G_= 0.0000832 Group: η^2^_G_= 0.048 Interaction: η^2^_G_= 0.004 | Mean and SD | Baseline | Control | 2.456 ±0.2914 |
|  |  |  |  |  |  |  |  |  |  | Functionnal | 2.494 ±0.0913 |
|  |  |  |  |  |  |  |  |  | No Stimulation | Control | 2.456 ±0.2914 |
|  |  |  |  |  |  |  |  |  |  | Functionnal | 2.544 ±0.0547 |
|  |  |  |  |  |  |  |  |  | Stimulation | Control | 2.478 ±0.0947 |
|  |  |  |  |  |  |  |  |  |  | Functionnal | 2.527 ±0.0733 |
| Supplementary Figure S4C | Paw withdrawal duration after Formalin intraplantar injection (1st phase 0-10min) | Unpaired t-test with Welch correction | Functionnal=9 Control=10 | t_(15.09)_ =0.7923 | p=0.4405 | ns | Cohen d = -0.353 | Mean and SD | Control | 122.7 ±44.87 |  |
|  |  |  |  |  |  |  |  |  | Functionnal | 141.6 ±57.79 |  |
|  | Paw withdrawal duration after Formalin intraplantar injection (2nd phase 10-60min) | Unpaired t-test with Welch correction | Functionnal=9 Control=10 | t_(16.98)_ =0.1144 | p=0.9102 | ns | Cohen d = 0.0498 | Mean and SD | Control | 224.4 ±131.5 |  |
|  |  |  |  |  |  |  |  |  | Functionnal | 217.9 ±113.2 |  |
| Supplementary Figure S4D | Thermal sensitivity evoked with radiant heat beam (Non-Implanted Right paw) | Unpaired t-test with Welch correction | Functionnal=11 Control=11 | t_(15.13)_ =0.4833 | p=0.6358 | ns | Cohen d = -0.199 | Mean and SD | Control | 7.405 ± 1.618 |  |
|  |  |  |  |  |  |  |  |  | Functionnal | 7.672 ±0.8502 |  |
